# Cardiovascular health in breast cancer patients: insight on BRCA1/2 mutations impact

**DOI:** 10.1186/s40959-025-00302-z

**Published:** 2025-01-21

**Authors:** Panagiotis Mallios, Mehdi Dehghan Manshadi, Laila Hubbert, Tajeri Aydin, Claudia Maes, Johanna Rantala, Annelie Liljegren, Narsis A. Kiani, Elham Hedayati

**Affiliations:** 1 Department of Cardiology, Department of Health, Medicine and Caring Sciences, Linkoping University, Norrkoping, Sweden; 2 Algorithmic Dynamics Lab, Center of Molecular Medicine, Karolinska Institute, Stockholm, Sweden; 3 Centre for Clinical Cancer Studies, Karolinska University Hospital, Stockholm, Sweden; 4 Department of Molecular Medicine and Surgery, Karolinska Institutet, Stockholm, Sweden; 5 Department of Clinical Genetics, Karolinska University Hospital, Stockholm, Sweden; 6 Department of Medicine, South General Hospital, Stockholm, Sweden; 7 Department of Oncology-Pathology, Karolinska Institutet, Stockholm, SE-171 77 Sweden; 8 Department of Oncology, South General Hospital, Stockholm, Sweden

**Keywords:** Breast Neoplasms/ complications, BRCA1 protein, BRCA2 protein, Cardio-oncology, Cardiovascular disease, Risk factors, Retrospective studies

## Abstract

**Background:**

Breast cancer (BC) and cardiovascular disease (CVD) are prevalent comorbidities in aging populations. Advances in BC treatment have improved survival rates but increased the risk of CVD, particularly among younger patients with BRCA1/2 mutations. BRCA1/2 gene mutations, prevalent in younger BC patients, impair cardioprotective effects, elevating CVD risk alongside cancer treatments. This study examined the prevalence and incidence of CVD and cardiovascular risk factors (CVRFs) before and after BC diagnosis in BRCA1/2 mutation carriers (BRCA-BC) and those with sporadic BC (Sporadic-BC).

**Methods:**

This descriptive retrospective cohort study analyzed BC patients from 1995 to 2020 in Stockholm-Gotland, Sweden. Data from regional and national registries provided insights into CVRFs, pre-existing CVDs, demographics, and cancer treatments. Analyses focused on single and multiple CVD events, comparing inpatient and outpatient settings across subgroups.

**Results:**

The cohort included 438 BRCA-BC and 32,626 Sporadic-BC patients. BRCA-BC patients were younger at BC diagnosis (median: 45 years, IQR 37–53) and first CVD event (median: 62 years, IQR 53–68) compared to Sporadic-BC patients (median: 61 years, IQR 51–71; and 74 years, IQR 65–81, respectively). Before BC diagnosis, CVD prevalence was lower in BRCA-BC patients (4.2%) than in Sporadic-BC patients (11.1%). Post-diagnosis, CVD prevalence increased in both groups, reaching 19.7% in BRCA-BC and 24.6% in Sporadic-BC patients. Heart failure (HF) was the most common major adverse cardiovascular event (MACE), affecting 4.6% of BRCA-BC and 9.5% of Sporadic-BC patients. Sporadic-BC patients exhibited a higher overall cardiovascular burden, including arrhythmias, coronary artery disease, and stroke.

**Conclusions:**

Distinct cardiovascular profiles between BRCA-BC and Sporadic-BC patients underscore the need for tailored survivorship care. Early cardiovascular screening benefits BRCA-BC patients, while Sporadic-BC patients require comprehensive management of pre-existing CVRFs. These findings align with international cardio-oncology guidelines advocating integrated cardiovascular care for BC survivors.

**Supplementary Information:**

The online version contains supplementary material available at 10.1186/s40959-025-00302-z.

## Introduction

Breast cancer (BC) and cardiovascular disease (CVD) are a significant burden as the population ages. In Sweden, BC ranks as the most prevalent malignant disease among females, accounting for more than 8,000 cases annually, with a recent mortality rate of approximately 15%. Early detection and treatment advances have contributed to a decrease in BC mortality in recent decades [1–4].

Most BC cases occur sporadically, driven by environmental, lifestyle, and random genetic factors without a known genetic predisposition (sporadic BC). In contrast, hereditary BC is associated with specific genetic mutations, such as *BRCA1* or *BRCA2* mutations, which account for 5–10% of BC cases but are present in only 0.2% of the general population [5, 6].

*BRCA1* and *BRCA2* genes, known for their roles in tumor suppression and DNA repair, may also influence cardiovascular health through enhanced endothelial repair and reduced oxidative stress [7]. Preclinical studies highlight the importance of functional BRCA1/2 in protecting cardiomyocytes and vascular integrity, including resistance to doxorubicin-induced DNA damage and promoting endothelial function. However, mutations in these genes impair DNA repair pathways, which may increase susceptibility to cardiotoxicity, particularly during cancer treatments like anthracyclines [7]. However, further research is necessary to substantiate these cardioprotective mechanisms and their clinical relevance. Individuals with *BRCA1/2* mutations typically develop BC at a younger age, with a median diagnosis age of 45 compared to 66 years for sporadic BC [6].

CVD and cancer share several risk factors, including age, smoking, hypertension, diabetes, hyperlipidemia, chronic kidney disease, and obesity. These risk factors are mediated by common pathophysiological mechanisms such as inflammation, oxidative stress, and immune dysfunction [8]. Additionally, cancer treatments—such as radiotherapy to the left chest and systemic therapies, including anthracycline-based chemotherapy and targeted treatments like Human Epidermal Growth Factor Receptor 2 (HER2) inhibitors—can lead to cancer therapy-related cardiovascular toxicity (CTR-CVT) [9, 10]. The risk of CTR-CVT increases with age, pre-existing cardiovascular risk factors (CVRFs), and comorbidities, and it can occur during or after cancer treatment [11–14]. Given the shared risk factors and treatment-related toxicities contributing to cardiovascular morbidity, understanding CVD patterns in BC subtypes is critical for developing targeted survivorship care strategies.

CVD encompasses a variety of conditions, such as angina pectoris and atrial fibrillation/flutter (AF). Severe cardiovascular events, referred to as Major Adverse Cardiovascular Events (MACE), are a subset of CVD that includes myocardial infarction (MI), stroke, and hospitalization for heart failure (HF). These events signify severe cardiovascular outcomes that lead to increased morbidity and mortality, particularly in patients treated with potentially cardiotoxic cancer therapies [15]. These events are essential for assessing the severity of CTR-CVT and understanding the lifelong cardiovascular burden faced by cancer survivors [16, 17].

Despite these challenges, there is a lack of prospective clinical data on the prevalence and outcomes of CVD among Swedish females with BRCA1/2 mutations who develop BC. This study aims to investigate the prevalence and incidence of CVRF and CVD before and after BC diagnosis in women with BRCA1/2 mutations (BRCA-BC) and Sporadic-BC. Additionally, it seeks to explore the sequence of CVD occurrences following BC diagnosis to evaluate the cardiovascular burden in these patient groups. Using 25 years of registry data, this descriptive study analyzes cardiovascular morbidity and risk factors among women with Sporadic-BC and BRCA-BC in the Stockholm-Gotland region, focusing on BRCA1/2 mutation carriers.

## Methods

This study was a register-based cohort analysis of all females diagnosed with BC, both with and without confirmed BRCA mutations, in the Stockholm-Gotland healthcare region (population: 2.5 million), Sweden, from January 1, 1995, to December 31, 2020. The Regional Ethics Review Board at the Karolinska Institute approved this study (2018/2669-31/2), and the study adhered to the STROBE guidelines and the Declaration of Helsinki. According to Swedish legislation, patients included in national quality registries do not need to provide written informed consent for their data to be used in healthcare research.

### Study population

The study included two groups: BRCA-BC and Sporadic-BC. The BRCA-BC group comprised females with confirmed BRCA1/2 mutations and a BC diagnosis, identified through genetic counseling at the Department of Clinical Genetics, Karolinska University Hospital (1995–2020), and subsequently linked to BC registries. The Sporadic-BC group included females diagnosed with BC without confirmed BRCA1/2 mutations.

The general female population consisted of all women aged 20 to 85 + who resided in the Stockholm region from 1998 (2001 for outpatients) to 2019. This population served as a baseline for comparing the prevalence and incidence of CVD across BRCA-BC, sporadic-BC, and the general population.

### Data sources

Data for the study were derived from several national and regional Swedish registries. The Swedish National Quality Register for BC provided clinical information for patients diagnosed between 2008 and 2020, while the Regional Quality Register for BC in Stockholm covered diagnoses between 1995 and 2007. These registries supplied details on BC diagnosis dates, menopausal status, tumor laterality, disease staging, and oncological treatments. Additional cancer diagnosis and staging data were retrieved from the Swedish Cancer Register [18]. The National Patient Register (NPR) was used to collect information on comorbidities and cardiovascular events based on International Classification of Diseases (ICD) codes (Supplemental Tables 1 and 2) [19, 20]. The NPR includes inpatient care data since 1987 and outpatient care data since 2001, although it excludes primary care data (Supplemental Table 1). Patients in each group were divided into four subgroups based on the timing of cardiovascular events (before or after BC diagnosis) and the care setting (inpatient or outpatient). Overlaps across these categories may result in summed numbers exceeding the total patient count. For patients listed in both inpatient and outpatient registries on the same date, only the inpatient data were retained to prevent double-counting. Events occurring on different dates were included in both groups to ensure comprehensive data capture, with overlaps accounted for in the corresponding tables.

General population data, including NPR data, were obtained from the Swedish Board of Health and Welfare’s statistical database, “Diagnoses in In-Patient and Specialised Open Care” [21].

### Inclusion and exclusion criteria

The study included females with a confirmed diagnosis of BC in either the BRCA-BC or Sporadic-BC groups. BRCA1/2 mutations were verified through genetic testing and counseling. Exclusion criteria applied to patients diagnosed with other neoplasms before their BC diagnosis, as well as those with stage 4 BC (distant metastases at diagnosis). Stage 4 BC cases were excluded due to the palliative nature of their treatment, which differs significantly in intent and outcomes compared to adjuvant or curative regimens.

### Cardiovascular risk factors and morbidity

CVRFs and CVD were identified using ICD codes (Supplemental Table 2). CVRFs included diagnoses of essential hypertension (I10), diabetes mellitus (E10, E11), hyperlipidemia (E78), obesity (E65, E66), chronic kidney disease (N18), and acute or unspecified renal failure (N17, N19). CVD was defined as one or more of the following conditions: angina pectoris (I20), arrhythmias including AF (I48), complete heart block (I44.2), and ventricular arrhythmias (ventricular tachycardia, I47.2; ventricular fibrillation/flutter, I49.0), claudication (I73.9), HF (I50), MI (ST-Elevation MI (STEMI), Non- ST-Elevation MI (NSTEMI), or subsequent MI; I21.0–I21.4), stroke (I63, I64). Cardiovascular interventions, including coronary stent implantation (Z95.5), coronary artery bypass grafting (CABG; Z95.1), pacemaker implantation (Z95.0), and resynchronization therapy or implantable cardioverter defibrillator (CRT/ICD; Z95.810), were also included in the definition of CVD.

MACE was defined as HF, MI, and stroke,

### Study outcomes

The primary outcomes were the prevalence and incidence of CVRF and CVD before and after a BC diagnosis in patients with or without a known BRCA mutation. The secondary outcome focused on the sequence of CVD occurrences following BC diagnosis.

### Statistical analysis

Patients were grouped into age categories (21–29, 30–39, 40–49, 50–59, 60–69, 70–79, and ≥ 80 years) and treatment eras (1995–2007 and 2008–2019 for BC; 1996–2007 and 2008–2020 for CVD) to account for changes in treatment guidelines (Supplemental Method), registry practices, and general health trends.

Descriptive statistics summarized patient characteristics and baseline data. Categorical variables were presented as numbers (n) and percentages (%), while continuous variables, given their skewed distributions, were summarized as medians and interquartile ranges (IQRs). Follow-up began on January 1, 1995, with the origin defined as the date of BC diagnosis (first cancer), and ended on December 31, 2020, or the date of death, whichever occurred first.

This analysis provided a detailed overview of cardiovascular morbidity in BC patients, particularly those with BRCA mutations. The key analyses included reporting patient characteristics and distributions across age groups and treatment eras; Calculating the incidence rates of CVRF and CVD before and after BC diagnosis in both the BRCA-BC and sporadic-BC groups; Examining the sequence and co-occurrence of single and multiple CVD events before and after BC diagnosis; Analyzing the number of patients with varying numbers of CVD events relative to BC diagnosis. Age distributions for BC cohorts (BRCA-BC and sporadic-BC) were compared with those of the general female population in the Stockholm region. General population data were collected at 5-year intervals for females aged 20 to 85 + years from 1998 (2001 for outpatients) to 2019. The same 5-year intervals were used for the BC cohorts, and event rates were normalized to cohort size per year and reported as rates per 100,000 individuals.

Age distributions for CVD were compared between BC cohorts and the general population using a non-parametric Kolmogorov-Smirnov test to evaluate differences.

For data querying, a comprehensive database was constructed using MySQL (Oracle Corporation, Redwood Shores, California, USA). MySQL scripts and MATLAB (MathWorks, Natick, Massachusetts, USA) were used for statistical analyses and visualizations to ensure precise data handling and visualization of cardiovascular patterns in the studied cohorts.

## Results

**Fig. 1:**
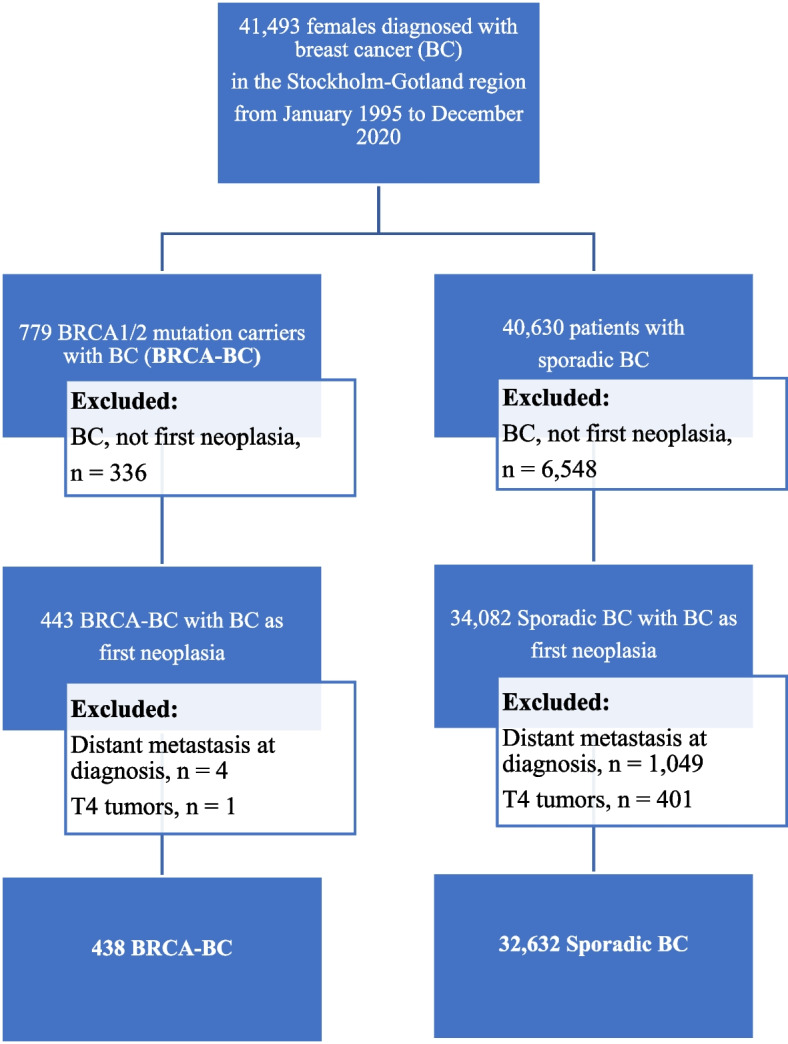
Flowchart

This 25-year observational study included 438 females with BRCA-BC and 32,626 with Sporadic-BC (Fig. 1). The median age at BC diagnosis was 16 years lower in BRCA-BC patients (45.0 years, IQR 37.0–53.0) than in Sporadic-BC patients (61.0 years, IQR 51.0–71.0). BRCA-BC patients were more frequently diagnosed with BC before age 40, with 35.9% diagnosed in this age group compared to 4.2% of Sporadic-BC patients (Table 1).

**Table 1:** Demographic and clinical characteristics of 33,154 female patients with Sporadic breast cancer (Sporadic BC) and BRCA1/2 carriers with BC (BRCA-BC), in in Stockholm-Gotland region, Sweden (January 1995 - December 2020)

	**Sporadic BC**	**BRCA-BC**
	32,626	438
**Median Age at BC (IQR)**	61.0 (51.00-71.00)	45.0 (37.00-53.00)
**BC in Age Groups years, No. (%)**		
21-29	122 (0.37)	25 (5.71)
30-39	1,249 (3.83)	132 (30.14)
40-49	5,119 (15.69)	131 (29.91)
50-59	8,168 (25.04)	90 (20.55)
60-69	8,694 (26.65)	45 (10.27)
70-79	5,648 (17.31)	12 (2.74)
80 ≤	3,626 (11.11)	3 (0.68)
**Year of BC Diagnosis No. (%)**		
1995 – 2007	15,392 (47.18)	205 (46.80)
2008 – 2019	17,221 (52.78)	233 (53.20)
2020 ≤	13 (0.04)	0 (0.00)
**Diagnosed with CVD, No. (%)**	8,603 (26.37)	43 (9.81)
**Age at CVD years, median (IQR)**	74.0 (65.00-81.00)	62.0 (53.00-68.00)
**First CVD in Age Groups, No. (age median)**		
10-20	1(19.0)	0 (-)
21-29	4 (26.0)	0 (-)
30-39	36 (36.0)	0 (-)
40-49	235 (46.0)	5 (44.0)
50-59	903 (56.0)	12 (53.0)
60-69	1,981 (66.0)	17 (64.0)
70-79	2,893 (75.0)	6 (73.5)
80 ≤	2,550 (85.0)	3 (84.0)
**Year of First CVD Diagnosis, No. (%)**		
1996-2007	3,371 (10.33)	12 (2.74)
2008-2020	4.596 (14.09)	31 (7.08)
**Time from First CVD Diagnosis to BC Diagnosis years, median (IQR)**	5.45 (2.31-9.66)	4.70 (3.39-5.70)
**Time from BC Diagnosis to First CVD Diagnosis. Years, median (IQR)**	6.15 (2.20-11.47)	10.90 (6.54-15.43)
**Menstruation Status at BC Diagnosis** ^a^**, No. (%)**		
Premenopausal	8,284 (25.39%)	258 (58.90%)
Perimenopausal	14,850 (45.52%)	87 (19.86%)
Postmenopausal	8,236 (25.24%)	27 (6.16%)
Unknown	1,030 (3.16%)	10 (2.28%)
Missing	226 (0.69%)	56 (12.79%)
**Cardiovascular Risk Factors, n (%)**		
***Prior to BC Diagnosis***		
Essential hypertension outpatient	1,851 (5.67)	3 (0.68)
Essential hypertension inpatient	3,068 (9.40)	9 (2.05)
Diabetes mellitus outpatient	714 (2.19)	8 (1.83)
Diabetes mellitus inpatient	846 (2.59)	6 (1.37)
Hyperlipidemia outpatient	461 (1.41)	2 (0.46)
Hyperlipidemia inpatient	668 (2.05)	3 (0.68)
Obesity (BMI > 30 kg/m^2^) outpatient	381 (1.17)	7 (1.60)
Obesity (BMI > 30 kg/m^2^) inpatient	268 (0.82)	6 (1.37)
Chronic kidney disease outpatient	128 (0.39)	1 (0.23)
Chronic kidney disease inpatient	115 (0.35)	0 (-)
Acute/unspecified renal failure outpatient	25 (0.08)	0 (-)
Acute/unspecified renal failure inpatient	106 (0.32)	0 (-)
***After BC Diagnosis***		
Essential hypertension outpatient	4,870 (14.93)	49 (11.19)
Essential hypertension inpatient	7,484 (22.94)	53 (12.10)
Diabetes mellitus outpatient	1,636 (5.01)	14 (3.20)
Diabetes mellitus inpatient	2,090 (6.41)	18 (4.11)
Hyperlipidemia outpatient	846 (2.59)	5 (1.14)
Hyperlipidemia inpatient	1,410 (4.32)	8 (1.83)
Obesity (BMI > 30 kg/m^2^) outpatient	645 (1.98)	11 (2.51)
Obesity (BMI > 30 kg/m^2^) inpatient	791 (2.42)	15 (3.42)
Chronic kidney disease outpatient	450 (1.38)	2 (0.46)
Chronic kidney disease inpatient	844 (2.59)	4 (0.91)
Acute/unspecified renal failure outpatient	225 (0.69)	2 (0.46)
Acute/unspecified renal failure inpatient	858 (2.63)	6 (1.37)

*Abbreviations*: *CVD* Cardiovascular Disease, *BC* Breast Cancer, *IQR* Interquartile Range, *BMI* body mass index.

^a^Premenopausal: No menopausal transition. Perimenopausal: Transition within the first 5 years. Postmenopausal: Transition occurring after 5 years. Unsure: Status uncertain, e.g., due to hysterectomy or other reasons.

The prevalence of CVD was significantly higher in Sporadic-BC patients (24.4%) compared to BRCA-BC patients (9.8%). BRCA-BC patients experienced their first CVD at a median age of 12 years younger (62 years, IQR 53.0–68.0) than Sporadic-BC patients (74 years, IQR 65.0–81.0). Among those diagnosed with CVD, BRCA-BC patients experienced a longer median time from BC diagnosis to first CVD (10.9 years, IQR 6.5–15.4) compared to Sporadic-BC patients (6.2 years, IQR 2.2–11.5) (Table 1).

### Cardiovascular risk factors

The BRCA-BC group exhibited lower baseline CVRF rates compared to the Sporadic-BC group. However, both groups showed increases in CVRF prevalence after BC diagnosis. Essential hypertension was the most common CVRF, with rates rising from 2.1 to 12.1% (inpatient) and 0.7–11.2% (outpatient) in BRCA-BC patients and from 9.4 to 22.9% (inpatient) and 5.7–14.9% (outpatient) in Sporadic-BC patients. Obesity prevalence increased in both groups, from 1.4 to 3.4% (inpatient) and 1.6–2.5% (outpatient) in BRCA-BC patients and from 0.8 to 2.4% (inpatient) and 1.2–2.0% (outpatient) in Sporadic-BC patients. Similarly, diabetes mellitus rates rose from 1.4 to 4.1% (inpatient) and 1.8–3.2% (outpatient) in BRCA-BC patients and from 2.6 to 6.4% (inpatient) and 2.2–5.0% (outpatient) in Sporadic-BC patients (Table 1).

### Tumor biology and oncological treatments

Tumor stages and treatments varied between Sporadic-BC and BRCA-BC groups. Early-stage tumors (Cancer in situ, T0, Paget, T1) were more prevalent in Sporadic-BC patients (62.5%) compared to BRCA-BC patients (48.0%). Higher-stage tumors (T2 and T3) were more frequent in BRCA-BC patients (38.9%) compared to Sporadic-BC patients (36.5%). BRCA-BC patients were more likely to receive neo-adjuvant chemotherapy (85.4% vs. 8.4%), including anthracycline-containing regimens (28.8% vs. 16.5%), compared to Sporadic-BC patients. In contrast, Sporadic-BC patients were more commonly treated with radiotherapy (34.4% vs. 26.9%) and endocrine therapy (43.0% vs. 26.0%). HER2-inhibitor use was infrequent in both groups but slightly higher in Sporadic-BC patients (5.9% vs. 1.8%) (Table 2).

**Table 2:** BC Tumor biology characteristics and received oncological treatments of 32,626 with Sporadic BC and BRCA-BC, in Stockholm-Gotland region, Sweden (January 1995 - December 2020)

	**Sporadic BC**	**BRCA-BC**
	32,626	438
**BC Tumour Biology Characteristics, n (%)**		
***Laterality***		
Left breast	16,336 (50.07)	214 (48.86)
Right breast	15,485 (47.46)	211 (48.17)
Bilateral	803 (2.46)	13 (2.97)
Missing	2 (0.01)	0 (-)
***Tumour Stage***^a^		
Cancer in Situ	692 (2.12)	10 (2.28)
T0 and Paget	5,867 (17.98)	44 (10.05)
T1	13,844 (42.43)	156 (35.62)
T2	10,008 (30.67)	142 (32.42)
T3	1,913 (5.86)	30 (6.58)
TX	68 (0.21)	1 (0.23)
Unknown	224 (0.69)	2 (0.46)
Missing	10 (0.03)	53 (12.10)
***Lymph Node Stage*** ^b^		
0	28,372 (86.96)	301 (68.72)
1-3	4,050 (12.41)	81 (18.49)
NX	69 (0.21)	2 (0.46)
Missing	135 (0.41)	54 (12.33)
**Received Oncological Treatments** ^c^**, n (%)**		
***Treatment***		
***Neo-adjuvant Chemotherapy***		
Yes	2,752 (8.43)	374 (85.39)
No	29,874 (91.57)	64 (14.61)
***Adjuvant Chemotherapy***		
Yes	6,005 (18.41)	137 (31.28)
No	26,458 (81.09)	301 (68.72)
Missing	163 (0.50)	-
***Anthracycline Containing Chemotherapy***		
***Full-dose Anthracycline Given***		
Yes	5,375 (16.47)	126 (28.77)
No	27,251 (83.53)	312 (71.23)
***HER2-Inhibitor***		
Yes	1,915 (5.87)	8 (1.83)
No	30,711 (94.13)	430 (98.17)
***Endocrine Therapy***		
Yes	14,015 (42.96)	114 (26.03)
No	18,611 (57.04)	324 (73.97)
***Radiotherapy***		
Yes	11,217 (34.38)	118 (26.94)
No	21,279 (65.22)	314 (71.69)
Missing	130 (0.40)	6 (1.37)

*Abbreviations: CVD* Cardiovascular Disease, *BC* Breast Cancer, *HER2-Inhibitor* Human Epidermal Growth Factor Receptor 2 inhibitor

^a^Tumor stage categories: TX: Primary tumor cannot be assessed. T0: No evidence of a primary tumor; Tis: Carcinoma in situ (DCIS, or Paget disease of the breast with no associated tumor mass); T1: Tumor is 2 cm (3/4 of an inch) or less across; T2: Tumor is more than 2 cm but not more than 5 cm (2 inches) across. T3: Tumor is more than 5 cm across

^b^Lymph node stage categories: NX: Nearby lymph nodes cannot be assessed; N0: No cancer in nearby lymph nodes; N1: Cancer in 1-3 axillary lymph nodes or internal mammary nodes; N2: Spread to 4-9 lymph nodes or enlarged internal mammary nodes; N3: Extensive spread, involving 10 or more axillary nodes, collarbone nodes, or above the collarbone

^c^New variable from 2008 in the Swedish National Quality Register for BC

### Cardiovascular disease prevalence

**Fig. 2:**
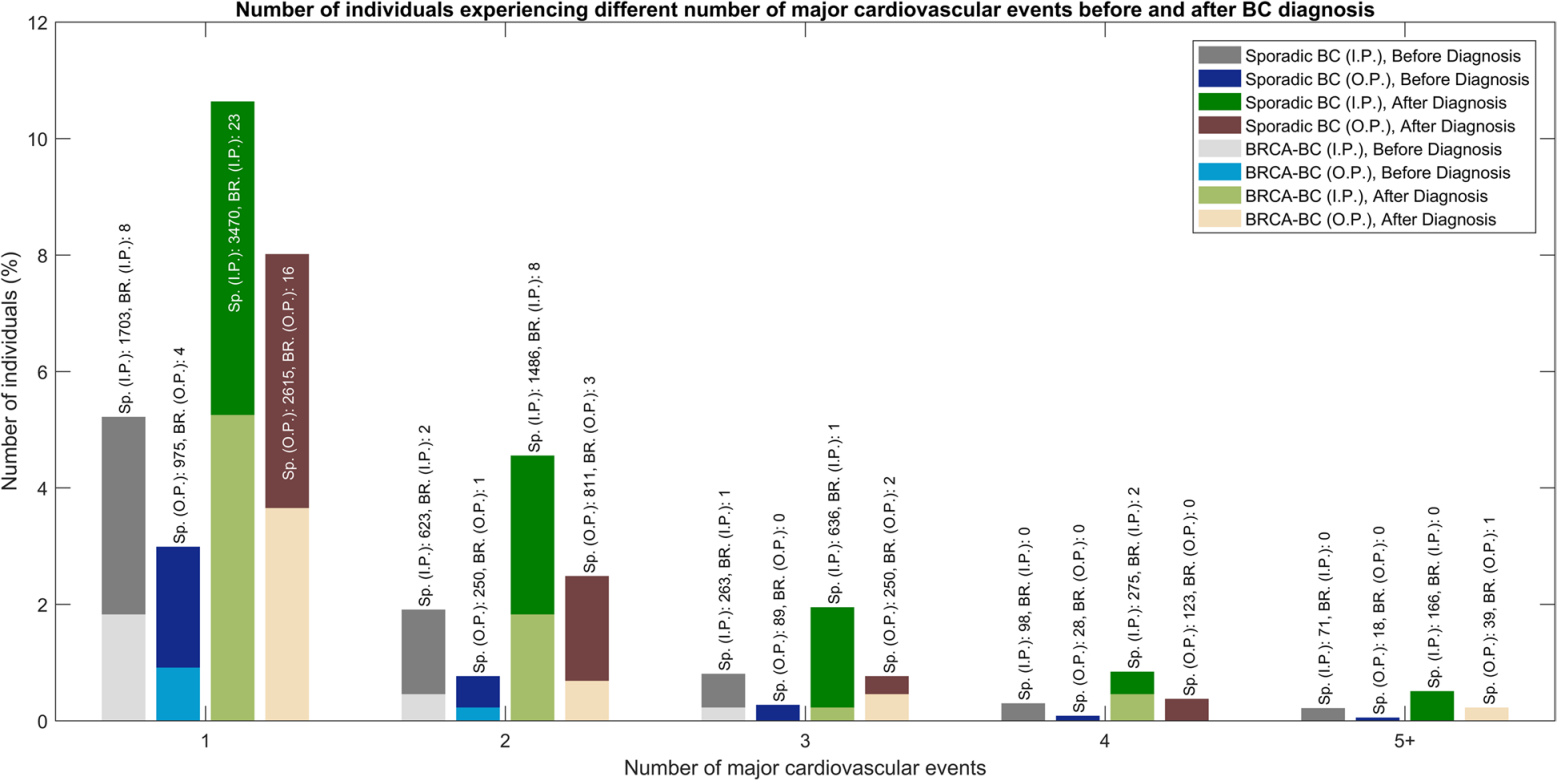
Comparison between the number of individuals experiencing different number of major cardiovascular events before and after BC diagnosis. Abbreviations are as follows: Sp.: Sporadic BC, BR.: BRCA-BC, I.P.: inpatient, and O.P.: outpatient

Sporadic-BC patients demonstrated consistently higher rates of CVD compared to BRCA-BC patients before and after BC diagnosis (Tables 3, 4, Fig. 2). Before BC diagnosis, the inpatient prevalence of CVD was 8.5% in Sporadic-BC patients, compared to 2.5% in BRCA-BC patients, while outpatient rates were 4.2% and 1.1%, respectively. Following BC diagnosis, CVD prevalence increased in both groups, reaching 18.5% (inpatient) and 11.8% (outpatient) in Sporadic-BC patients and 7.8% (inpatient) and 5.0% (outpatient) in BRCA-BC patients.

**Table 3:** Cardiovascular events prevalence from inpatient registry, median age at diagnosis, and time intervals between CVD and BC diagnosis in female patients with Sporadic breast cancer (Sporadic BC) and BRCA1/2 carriers with breast cancer (BRCA-BC), in Stockholm-Gotland region, Sweden (January 1995 - December 2020)

	**CVD Patients** (N (%))		**Median Age at CVD** (Years (IQR))		**Median Time Between CVD and BC** (Days (IQR))	
	**Sporadic BC**	**BRCA-BC**	**Sporadic BC**	**BRCA-BC**	**Sporadic BC**	**BRCA-BC**
**Before BC diagnosis**						
Any CVD	2,758 (8.45)	11 (2.51)	71.0 (62.00-79.00)	61.0 (53.50-66.00)	1,932.00 (829.25-3,480.75)	1544.00 (1,429.00-2,101.50)
Angina pectoris	1, 073 (3.29)	2 (0.46)	69.0 (60.00-77.00)	59.0 (57.00-61.00)	2,583.0 (1,262.00-4,427.00)	2,135.5 (1415.75-2855.25)
Atrial fibrillation or atrial flutter	989 (2.91)	1 (0.23)	75.0 (67.00-82.00)	64.0 (64.00-64.00)	1,332.0 (518.00-2,487.00)	1394.00 (1394.00-1394.00)
Heart Failure	930 (2.85)	2 (0.46)	77.0 (70.00-83.00)	67.0 (65.50-68.50)	1394 (533.25-2606.25)	1429.5 (1411.75-1447.25)
Stroke	638 (1.96)	3 (0.68)	73.0 (64.00-80.00)	61.0 (55.50-64.50)	1721 (689.75-3144.75)	2005.0 (1855.00-2114.00)
Myocardial Infarction (NSTEMI)	203 (0.62)	1 (0.23)	75.0 (63.00-81.00)	46.0 (46.00-46.00)	1520 (694.5-2614.5)	1544.0 (1544.00-1544.00)
Myocardial Infarction (STEMI)	83 (0.25)	1 (0.23)	70.0 (60.00-79.00)	61.0 (61.00-61.00)	1821 (965-3422)	1464.0 (1464.00-1464.00)
Complete heart block	48 (0.15)	2 (0.46)	78.5 (70.00-83.00)	72.5 (62.25-82.75)	1225.5 (882.25-2142.75)	1106.0 (560.00-1652.00)
Claudication	44 (0.13)	0 (0.00)	75.0 (69.75-80.00)	No patients	1050.0 (488.25-2381)	No patients
Ventricular tachycardia	42 (0.13)	0 (0.00)	64.0 (55.50-72.00)	N/A	1694.5 )1087.75-3977.25(	N/A
Ventricular fibrillation/flutter	4 (0.01)	0 (0.00)	53.0 (47.00-59.75)	N/A	3,023.0 (1,790.75-4,870.25)	N/A
Subsequent myocardial infarction	8 (0.02)	0 (0.00)	68.0 (59.75-77.25)	N/A	1361.0 (916.25-3840.75)	N/A
*Cardiovascular interventions:*						
Resynchronization therapy and implantable cardioverter defibrillator	149 (0.46)	1 (0.23)	75.0 (70.00-82.00)	58.0 (58.00-58.00)	1135.0 (491.00-2345.00)	1.0 (1.00-1.00)
Pacemaker implantation	148 (0.45)	1 (0.23)	75.0 (70.00-82.00)	58.0 (58.00-58.00)	1134.0 (488.50-2285.75)	1.0 (1.00-1.00)
Coronary artery bypass grafting	85 (0.26)	0 (0.00)	73.0 (67.00-77.00)	No patients	1561.0 (751.00-2564.00)	N/A
Coronary stent implantation	74 (0.23)	1 (0.23)	69.5 (62.00-76.75)	61.0 (61.00-61.00)	1574.5 (645.00-2575.75)	1463.0 (1463.00-1463.00)
**After BC diagnosis**						
Any CVD	6033 (18.49)	34 (7.76)	77.0 (70.00-84.00)	66.0 (58.25-72.00)	1892.0 (569.00-3806.00)	3662.5 (650.00-5512.00)
Atrial fibrillation or atrial flutter	2883 (8.84)	12 (2.74)	79.0 (72.00-85.00)	66.00 (63.00-75.75)	2472.0 (987.50-4416.00)	3921 (1588.75-5855.50)
Heart Failure	2711 (8.31)	12 (2.74)	81.0 (73.00-87.00)	65.5 (60.75-75.25)	2249.0 (856.50-4282.50)	4899.0 (3506.00-6069.50)
Stroke	1409 (4.32)	7 (1.60)	80.0 (72.00-86.00)	66.0 (58.50-68.50)	2369.0 (1156.00-4190.00)	3088.0 (1509.00-3728.00)
Angina pectoris	940 (2.88)	3 (0.68)	77.0 (69.00-83.00)	67.0 (62.00-69.50)	1464.5 (295.00-3164.75)	4983.0 (2547.00-5317.00)
Myocardial Infarction (NSTEMI)	497 (1.52)	3 (0.68)	78.0 (71.00-84.00)	74.0 (73.00-74.50)	2583.0 (1037.00-4314.00)	4985.0 (2837.00-5988.50)
Myocardial Infarction (STEMI)	171 (0.52)	0 (0.88)	77.0 (65.00-85.00)	N/A	2468.0 (864.50-4720.00)	N/A
Complete heart block	154 (0.47)	2 (0.46)	80.0 (73.00-87.75)	66.5 (62.75-70.25)	2640.50 (1301.25-4261.00)	3666.5 (2318.75-5014.25)
Claudication	157 (0.48)	0 (0.00)	77.0 (69.00-84.00)	N/A	2411.0 (847.00-4171.00)	N/A
Ventricular tachycardia	54 (0.17)	1 (0.23)	68.0 (59.00-76.75)	51.0 (51.00-51.00)	2041.5 (738.50-3568.75)	520.0 (520.00-520.00)
Ventricular fibrillation/flutter	19 (0.06)	0 (0.00)	76.0 (65.00-79.00)	N/A	2149.0 (856.00-5175.00)	N/A
Subsequent myocardial infarction	11 (0.03)	0 (0.00)	85.0 (80.50-87	N/A	1961.0 (1393.00-3547.00)	N/A
*Cardiovascular interventions:*						
Pacemaker implantation	497 (1.52)	5 (1.14)	79.0 (73.00-85.00)	75.0 (61.00-84.00)	2809.0 (1067.00-4756.00)	1245.0 (310.00-6108.00)
Resynchronization therapy and implantable cardioverter defibrillator	497 (1.52)	5 (1.14)	79.0 (73.00-85.00)	75.0 (61.00-84.00)	2809.0 (1067.00-4756.00)	1245.0 (310.00-6108.00)
Coronary stent implantation	229 (0.70)	0 (0.00)	74.0 (67.00-81.00)	N/A	3098.0 (1294.00-5311.00)	N/A
Coronary artery bypass grafting	138 (0.42)	0 (0.00)	74.0 (67.00-79.75)	N/A	2034.0 (841.00-3587.00)	N/A

*Abbreviations*: *CVD* Cardiovascular Disease, *BC* Breast Cancer, *IQR* Interquartile Range, *NSTEMI* Non-ST-Elevation Myocardial Infarction, *STEMI* ST-Elevation Myocardial Infarction, *N/A* Not Applicable

**Table 4:** Cardiovascular events prevalence from outpatient registry, median age at diagnosis, and time intervals in female patients with Sporadic breast cancer (Sporadic BC) and BRCA1/2 Carriers with Breast Cancer (BRCA-BC), in Stockholm-Gotland region, Sweden (January 1995 - December 2020)

	**CVD Patients** (N/NCP* (%))		**Median Age at CVD** (Years (IQR))		**Median Time Between CVD and BC** (Days (IQR))	
	Sporadic BC	BRCA-BC	Sporadic BC	BRCA-BC	Sporadic BC	BRCA-BC
Number of patients	32,626	438				
**Before BC diagnosis**						
Any CVD	1360/1010 (4.17)	5/4 (1.14)	68.0 (61.00-77.00)	61.0 (61.00-62.00)	1697.5 (741.75-2920.25)	1656.0 (1427.00-1904.00)
Atrial fibrillation or atrial flutter	645/587 (2.03)	2/2 (0.46)	70.0 (63.00-77.00)	63.0 (62.00-64.00)	1445.0 (652.00-2557.00)	868.0 (486.00-1250.00)
Angina pectoris	404/273 (1.24)	1/1 (0.23)	67.0 (59.00-74.00)	62.0 (62.00-62.00)	2125.5 (967.25-3469.25)	3842.0 (3842.00-3842.00)
Heart Failure	285/242 (0.87)	0/0 (0.00)	75.0 (65.00-82.00)	N/A	1260.0 (450.00-2200.00)	N/A
Stroke	158/122 (0.48)	2/2 (0.46)	68.0 (60.25-75.75)	56.0 (53.50-58.50)	1660.5 (664.50-2994.25)	1844.5 (1534.75-2154.25)
Claudication	50/24 (0.15)	0/0 (0.00)	70 (61.50-79.00)	N/A	656.5 (549.25-2635.25)	N/A
Ventricular tachycardia	34/13 (0.10)	0/0 (0.00)	57.0 (45.25-62.00)	N/A	1541.0 (787.50-2960.75)	N/A
Complete heart block	28/23 (0.09)	0/0 (0.00)	72.0 (65.50-78.25)	N/A	1337.0 (996.25-2494.25)	N/A
Myocardial Infarction (NSTEMI)	15/13 (0.05)	0/0 (0.00)	64 (58.50-72.00)	N/A	1271.0 (523.00-2398.00)	N/A
Myocardial Infarction (STEMI)	4/2 (0.01)	0/0 (0.00)	63.5 (60.75-69.75)	N/A	2481.0 (2075.50-3453.50)	N/A
Ventricular fibrillation/flutter	2/1 (0.01)	0/0 (0.00)	71.5 (70.75-72.25)	N/A	3183.5 (2306.25-4060.75)	N/A
Subsequent myocardial infarction	0 (0.00)	0/0 (0.00)	N/A	N/A	N/A	N/A
*Cardiovascular interventions:*						
Pacemaker implantation	96/92 (0.29)	0/0 (0.00)	72.0 (66.00-79.00)	N/A	1316.0 (504.00-2638.00)	N/A
Resynchronization therapy and implantable cardioverter defibrillator	96/92 (0.29)	0/0 (0.00)	72.0 (66.00-79.00)	N/A	1316.0 (504.00-2638.00)	N/A
Coronary stent implantation	76/45 (0.23)	1/1 (0.23)	67.0 (62.00-76.00)	61.0 (61.00-61.00)	1676.5 (594.00-2869.50)	1427.0 (1427.00-1427.00)
Coronary artery bypass grafting	56/47 (0.17)	0/0 (0.00)	70.0 (63.75-75.00)	N/A	1660.0 (840.50-2442.25)	N/A
**After BC diagnosis**						
Any CVD	3838/2894 (11.76)	22/17 (5.02)	74.0 (67.00-81.00)	62.0 (52.50-66.75)	2318.0 (896.75-4241.75)	3282.0 (1527.00-4990.75)
Atrial fibrillation or atrial flutter	1826/1526 (6.10)	5/5 (1.14)	76.0 (69.00-82.00)	68.0 (63.00-68.00)	2743.0 (1181.25-4588.50)	2824.0 (2388.00-5304.00)
Heart Failure	1432/1092 (4.34)	10/7 (2.28)	76.0 (69.00-84.00)	61.5 (51.75-68.25)	2710.5 (1007.75-4809.25)	4715.0 (3647.50-5293.75)
Stroke	482/388 (1.48)	4/4 (0.91)	75.0 (68.00-82.00)	67.5 (60.25-70.50)	2563.0 (1212.00-4309.75)	3340.0 (2421.50-4929.25)
Angina pectoris	623/379 (1.91)	3/1 (0.68)	72.0 (66.00-80.00)	57.0 (50.50-61.00)	2189.0 (949.50-3888.00)	1334.0 (738.00-2389.00)
Claudication	163/66 (0.44)	0/0 (0)	74.0 (69.75-80.00)	N/A	2760.0 (1369.50-4795.00)	N/A
Myocardial Infarction (NSTEMI)	58/43 (0.18)	1/1 (0.23)	74.0 (67.50-78.75)	76.0 (76.00-76.00)	3212.5 (1623.25-4748.00)	1392.0 (1392.00-1392.00)
Myocardial Infarction (STEMI)	25/22 (0.08)	0/0 (0)	69.0 (63.00-80.00)	N/A	2870.0 (937.00-4894.00)	N/A
Complete heart block	76/52 (0.23)	3/2 (0.68)	77.5 (72.75-83.00)	62.0 (60.50-65.00)	2376.0 (1287.50-4301.75)	389.0 (236.00-2310.00)
Ventricular tachycardia	47/23 (0.14)	1/0 (0.23)	66.0 (59.00-70.00)	67.00 (67.00-67.00)	2909.0 (1222.50-3856.50)	2478.0 (2478.00-2478.00)
Myocardial Infarction (STEMI)	25/22 (0.08)	0/0 (0)	69.0 (63.00-80.00)	N/A	2870.0 (937.00-4894.00)	N/A
Ventricular fibrillation/flutter	7/3 (0.02)	0/0 (0)	62.0 (59.00-69.50)	N/A	4299.0 (2433.00-4838.00)	N/A
Subsequent myocardial infarction	2/0 (0.01)	0/0 (0)	64.0 (62.50-65.50)	N/A	639.5 (431.75-847.25)	N/A
*Cardiovascular interventions:*						
Pacemaker implantation	312/259 (0.96)	3/3 (0.68)	77.0 (71.00-82.00)	62.0 (60.50-62.00)	2652.0 (1052.50-4751.75)	389.0 (233.50-1247.50)
Resynchronization therapy and implantable cardioverter defibrillator	312/259 (0.96)	3/3 (0.96)	77 (71.00-82.00)	62.0 (60.50-62.00)	2652.0 (1025.50-4751.75)	389.0 (233.50-1247.50)
Coronary stent implantation	226/125 (0.69)	0/0 (0)	72.0 (66.00-77.00)	N/A	3234.0 (1823.25-5158.50)	N/A
Coronary artery bypass grafting	101/66 (0.31)	0/0 (0)	73.0 (66.00-78.00)	N/A	2358.0 (1063.00-3858.00)	N/A

NCP*: Total cases recorded in both inpatient and outpatient registries. Patients with entries in both registries on the same date are classified as inpatients

*Abbreviations*: *CVD* Cardiovascular Disease, *BC* Breast Cancer, *IQR* Interquartile Range, *NSTEMI* Non-ST-Elevation Myocardial Infarction, *STEMI* ST-Elevation Myocardial Infarction, *N/A* Not Applicable

### Age and time to first CVD

BRCA-BC patients experienced CVD at younger ages than Sporadic-BC patients. Before BC diagnosis, the median age at first CVD was 61 years (inpatient and outpatient) in BRCA-BC patients, compared to 71 years (inpatient) and 68 years (outpatient) in Sporadic-BC patients. After BC diagnosis, these trends persisted, with BRCA-BC patients developing CVD at a median age of 66 years (inpatient) and 62 years (outpatient), compared to 77 years (inpatient) and 74 years (outpatient) in Sporadic-BC patients. The time from BC diagnosis to first CVD was longer in BRCA-BC patients (median: 3,662.5 days inpatient; 3,282 days outpatient) than in Sporadic-BC patients (median: 1,892 days inpatient; 2,318 days outpatient).

### Cardiovascular subtypes

#### Atrial fibrillation/flutter and heart failure

AF and HF were the most prevalent cardiovascular conditions in both groups, particularly after BC diagnosis. In the inpatient setting, HF affected 8.3% of Sporadic-BC patients compared to 2.7% of BRCA-BC patients. AF was observed in 8.8% of Sporadic-BC patients and 2.7% of BRCA-BC patients. Similar trends were seen in the outpatient setting, with HF and AF affecting 4.3% and 6.1% of Sporadic-BC patients, compared to 1.6% and 1.1% of BRCA-BC patients, respectively.

#### Stroke

Stroke was more frequent in Sporadic-BC patients compared to BRCA-BC patients. In the inpatient setting, 4.3% of Sporadic-BC patients experienced a stroke, compared to 1.6% of BRCA-BC patients. Outpatient stroke rates were 1.5% in Sporadic-BC patients and 0.9% in BRCA-BC patients.

#### Ischemic heart disease

Ischemic heart disease, encompassing MI and angina pectoris, was also more common in Sporadic-BC patients. In the inpatient setting, MI was reported in 1.5% of Sporadic-BC patients, compared to 0.7% of BRCA-BC patients. Outpatient MI rates were 0.5% in Sporadic-BC patients, with no cases reported in BRCA-BC patients. Angina pectoris occurred in 2.9% (inpatient) and 1.9% (outpatient) of Sporadic-BC patients, compared to 0.7% (inpatient) and 0.2% (outpatient) in BRCA-BC patients.

#### Multiple CVD events

Sporadic-BC patients experienced a higher burden of multiple CVD events compared to BRCA-BC patients, both before and after BC diagnosis. In the inpatient setting, before BC diagnosis, 5.2% of Sporadic-BC patients experienced one event, 1.9% had two events, and 1.1% experienced three or more events (Fig. 2). After BC diagnosis, these rates increased to 10.6% with one event, 4.6% with two events, and 3.3% with three or more events. For BRCA-BC patients, the rates were substantially lower before and after BC diagnosis, with no cases of five or more events observed. This pattern was consistent in outpatient settings, as shown in Fig. 2.

#### Cardiovascular event after BC treatment

Sporadic-BC patients had higher HF incidence across all treatment groups compared to BRCA-BC patients. Without systemic therapy, HF occurred in 1,742 Sporadic-BC patients (median age: 82 years) and 11 BRCA-BC patients (median age: 64 years). Combined anthracycline and HER2-inhibitor therapy resulted in HF in 36 Sporadic-BC patients (median age: 64.5 years) but no cases in BRCA-BC patients. In the outpatient registry, HF was observed in 1,039 Sporadic-BC patients (median age: 78 years) and 8 BRCA-BC patients (median age: 58.5 years). The shortest time to HF onset occurred in Sporadic-BC patients receiving HER2-inhibitor therapy only (337 days outpatient, 702.5 days inpatient). Across all groups, BRCA-BC patients developed HF at a younger age (Table 5)

**Table 5:** The incidence heart failure after the BC diagnosis based on systemic anthracycline treatment and HER2-inhibitor , median age at diagnosis, and time intervals in female patients with Sporadic breast cancer (Sporadic BC) and BRCA1/2 carriers with breast cancer (BRCA-BC), in Stockholm-Gotland region, Sweden (January 2008 - December 2020)

	**HF Patients** (N)		**Median Age at HF** (IQR)		**Median Time Between BC Diagnosis** **and HF** (Days, IQR)	
	**Sporadic BC**	**BRCA-BC**	**Sporadic BC**	**BRCA-BC**	**Sporadic BC**	**BRCA-BC**
**Heart Failure Hospitalized (Inpatient):**						
No Anthracycline or HER2-Inhibitor	1,742	11	82.0 (75.00-88.00)	64.0 (59.50-72.00)	3,470.5 (1,682.00-5,203.00)	5,198.0 (4,090.50-6,153.00)
Anthracycline	88	1	67.0 (62.75-74.00)	76.0 (76.00-76.00)	1,138.5 (290.25-2,100.25)	1,388.0 (1,388.00-1,388.00)
HER2-Inhibitor	14	0	80.5 (71.00-83.00)	N/A	702.5 (177.50-1692.75)	N/A
Anthracycline + HER2-Inhibitor	36	0	64.5 (56.00-74.25)	N/A	601.0 (291.50-1414.25)	N/A
**Heart Failure (Outpatient):**						
No Anthracycline or HER2-Inhibitor	1,039/72*	8/5*	78.0 (71.00-85.00)	58.5 (51.00-66.75)	3,779.0 (1,875.50-5,440.00)	4,946.5 (4,459.75-5,431.50)
Anthracycline	80/51*	1/1*	67.0 (58.75-74.00)	76.0 (76.00-76.00)	1,043.5 (356.5-1,977.50)	1,317.0 (1,317.00-1,317.00)
HER2-Inhibitor	19/9*	0/0*	70.0 (64.50-79.00)	N/A	337.0 (100.50-608.50)	N/A
Anthracycline + HER2-Inhibitor	72/23*	0/0*	61.5 (53.50-69.00)	N/A	410.5 (303.25-656.75)	N/A

NCP*: Total cases recorded in both inpatient and outpatient registries. Patients with entries in both registries on the same date are classified as inpatients

*Abbreviations*: *CVD* Cardiovascular Disease, *BC* Breast Cancer, *HER2-Inhibitor* Human Epidermal Growth Factor Receptor 2 inhibitor, *N/A* Not Applicable

Stroke incidence was substantially higher in Sporadic-BC patients across all treatment groups compared to BRCA-BC patients. Without systemic therapy, inpatient stroke occurred in 787 Sporadic-BC patients (median age: 80 years, IQR 72.00–88.00) and 4 BRCA-BC patients (median age: 62.5 years, IQR 47.00–70.50). Endocrine therapy combined with chemotherapy resulted in stroke in 37 Sporadic-BC patients (median age: 68 years, IQR 61.50–72.50) and 1 BRCA-BC patient (median age: 68 years). Outpatient records revealed younger median ages for BRCA-BC patients (e.g., 66 years with no therapy) compared to Sporadic-BC patients (77 years) (Table 6).

**Table 6:** The incidence stroke after the BC diagnosis based on systemic endocrine therapy and chemotherapy, median age at diagnosis, and time intervals in female patients with Sporadic breast cancer (Sporadic BC) and BRCA1/2 carriers with breast cancer (BRCA-BC), in Stockholm-Gotland region, Sweden (January 2008 - December 2020)

	**Stroke Patients (**N)		**Median Age at Stroke** (IQR)		**Median Time Between BC Diagnosis** **and Stroke** (Days, IQR)	
	**Sporadic BC**	**BRCA-BC**	**Sporadic BC**	**BRCA-BC**	**Sporadic BC**	**BRCA-BC**
**Stroke hospitalized (Inpatient):**						
No Endocrine or Chemotherapy	787	4	80.0 (72.00-88.00)	62.5 (47.00-70.50)	3920.0 (2230.00-5406.00)	3309.5 (853.75-4983.00)
Endocrine Therapy	160	0	79.0 (71.00-85.75)	N/A	1392.0 (621.50-2306.75)	N/A
Chemotherapy	13	0	70.0 (65.50-76.00)	N/A	589.0 (230.50-1149.00)	N/A
Endocrine + Chemotherapy	37	1	68.0 (61.50-72.50)	68.0 (68.00-68.00)	1587.0 (806.50-2052.50)	178.0 (178.00-178.00)
**Stroke (Outpatient):**						
No Endocrine or Chemotherapy	294/227*	3/3*	77.0 (70.00-84.00)	66.0 (43.00-75.00)	4005.5 (2253.5-5446.5)	3560.0 (3120.00-9037.00)
Endocrine Therapy	78/55*	0/0*	75.0 (69.00-83.00)	N/A	1229.5 (402.00-2246.50)	N/A
Chemotherapy	7/4*	0/0*	72.0 (60.00-76.00)	N/A	1391.0 (739.00-1868.00)	N/A
Endocrine + Chemotherapy	17/14*	1/1*	65.0 (55.00-68.00)	69.0 (69.00-69.00)	1520.0 (706.00-1849.50)	326.0 (326.00-326.00)

NCP*: Total cases recorded in both inpatient and outpatient registries. Patients with entries in both registries on the same date are classified as inpatients

*Abbreviations*: *CVD* Cardiovascular Disease, *BC* Breast Cancer, *N/A* Not Applicable

STEMI and NSTEMI were reported only in Sporadic-BC patients. STEMI occurred in 83 patients without systemic therapy (median age: 80 years, IQR 72.00–87.00) and 10 patients treated with radiotherapy (median age: 69.5 years, IQR 57.75–81.00). No STEMI cases were observed in BRCA-BC patients (Table 7). Similarly, NSTEMI was more frequent in Sporadic-BC patients, with median ages ranging from 65 years (radiotherapy + chemotherapy) to 79 years (no systemic therapy) (Table 8).

**Table 7:** The Incidence ST Elevation Myocardial Infarction (STEMI) after the BC diagnosis based on radiotherapy and systemic chemotherapy, median age at diagnosis, and time intervals in female patients with Sporadic breast cancer (Sporadic BC) and BRCA1/2 carriers with breast cancer (BRCA-BC), in Stockholm-Gotland region, Sweden (January 2008 - December 2020)

	**STEMI Patients (**N)		**Median Age at STEMI** (IQR)		**Median Time Between BC Diagnosis** **and STEMI** (Days, IQR)	
	**Sporadic BC**	**BRCA-BC**	**Sporadic BC**	**BRCA-BC**	**Sporadic BC**	**BRCA-BC**
**STEMI hospitalized (Inpatient):**						
No Radiotherapy or Chemotherapy	83	0	80.0 (72.00-87.00)	N/A	4772.0 (3128.00-5586.00)	N/A
Radiotherapy	10	0	69.5 (57.75-81.00)	N/A	336.0 (230.50-947.50)	N/A
Chemotherapy	0	0	No patients	N/A	No patients	N/A
Radiotherapy + Chemotherapy	7	0	73.0 (65.00-82.00)	N/A	878.0 (563.00-3157.00)	N/A
**STEMI (Outpatient):**						
No Radiotherapy or Chemotherapy	14/12*	0/0*	69.0 (58.75-80.50)	N/A	4692.5 (2850.25-5516.00)	N/A
Radiotherapy	4/4*	0/0*	67.0 (60.50-79.50)	N/A	369.5 (151.50-1377.25)	N/A
Chemotherapy	0/0*	0/0*	No patients	N/A	No patients	N/A
Radiotherapy + Chemotherapy	4/2*	0/0*	65.0 (47.75-77.75)	N/A	2059.0 (466.75-3193.00)	N/A

NCP*: Total cases recorded in both inpatient and outpatient registries. Patients with entries in both registries on the same date are classified as inpatients

*Abbreviations*: *CVD* Cardiovascular Disease, *BC* Breast Cancer, *N/A* Not Applicable

**Table 8:** The Incidence Non-ST Elevation Myocardial Infarction (NSTEMI) after the BC diagnosis based on radiotherapy and systemic chemotherapy, median age at diagnosis, and time intervals in female patients with Sporadic breast cancer (Sporadic BC) and BRCA1/2 carriers with breast cancer (BRCA-BC), in Stockholm-Gotland region, Sweden (January 2008 - December 2020)

	**NSTEMI Patients (**N)		**Median Age at NSTEMI** (IQR)		**Median Time Between BC Diagnosis** **and NSTEMI** (Days, IQR)	
	**Sporadic BC**	**BRCA-BC**	**Sporadic BC**	**BRCA-BC**	**Sporadic BC**	**BRCA-BC**
**NSTEMI hospitalized (Inpatient):**						
No Radiotherapy or Chemotherapy	279	2	78.0 (71.00-85.00)	73.5 (72.00-75.00)	3916.0 (2445.00-5594.00)	5988.5 (4985.00-6992.00)
Radiotherapy	28	0	75.0 (68.50-80.50)	N/A	1663.0 (319.50-2510.25)	N/A
Chemotherapy	5	0	82.0 (71.50-84.00)	N/A	1016.0 (526.00-2824.50)	N/A
Radiotherapy + Chemotherapy	20	1	70.0 (66.00-73.00)	74.0 (74.00-74.00)	1242.0 (407.25-2179.00)	689.0 (689.00-689.00)
**NSTEMI (Outpatient):**						
No Radiotherapy or Chemotherapy	39/30*	0	74.0 (70.00-79.00)	N/A	3722.0 (2899.00-5614.00)	N/A
Radiotherapy	11/7*	0	73.0 (67.00-75.00)	N/A	1375.0 (343.00-2622.00)	N/A
Chemotherapy	1/0*	0	53.0 (53.00-53.00)	N/A	1963.0 (1963.00-1963.00)	N/A
Radiotherapy + Chemotherapy	4/2*	1/1*	67.5 (52.75- 80.00)	74.0 (74.00-74.00)	2098.5 (406.00-3101.00)	689.0 (689.00-689.00)

NCP*: Total cases recorded in both inpatient and outpatient registries. Patients with entries in both registries on the same date are classified as inpatients

*Abbreviations*: *CVD* Cardiovascular Disease, *BC* Breast Cancer, *N/A* Not Applicable

### Comparison with the general female population

**Fig. 3:**
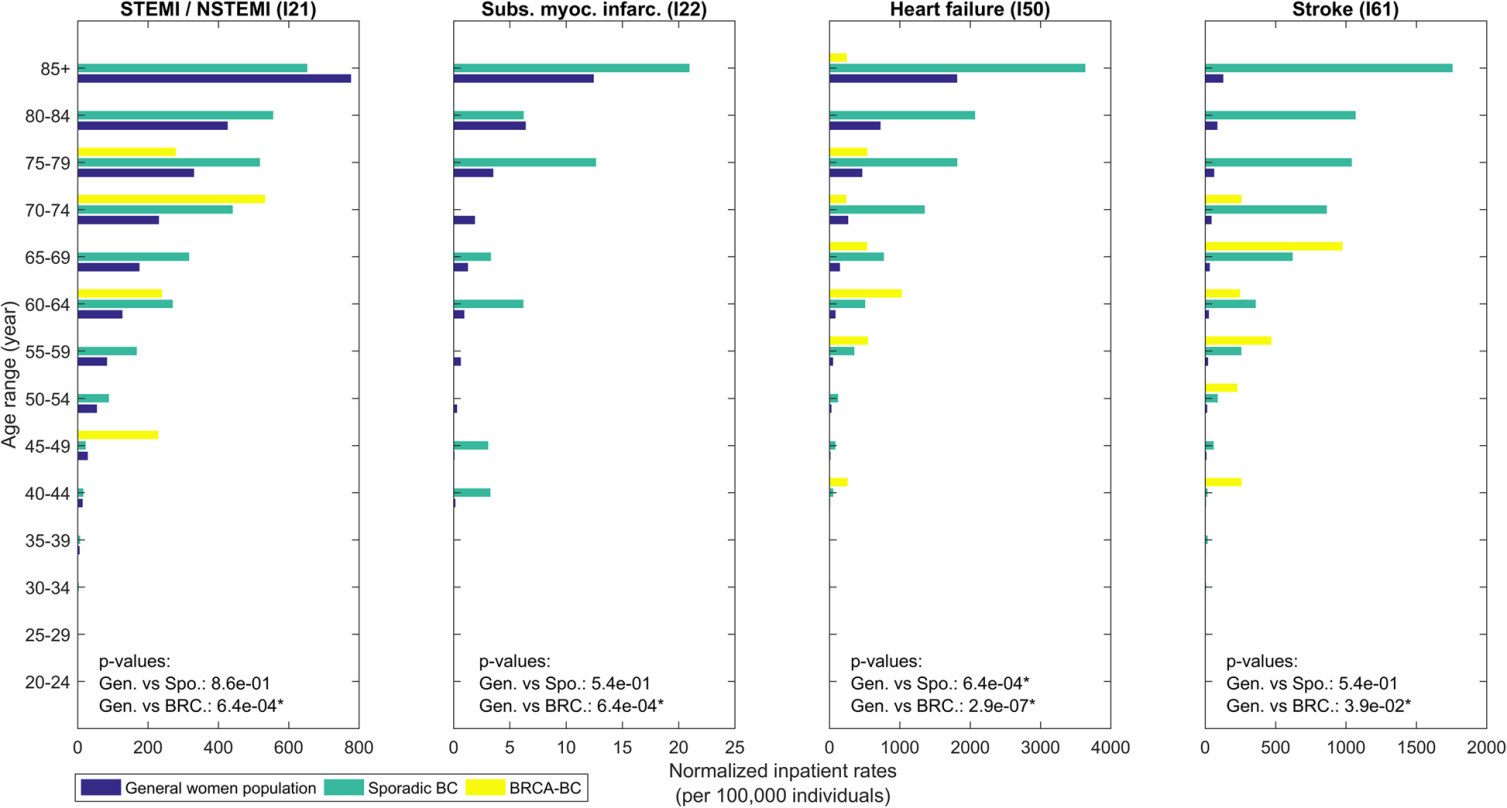
The distribution of normalized inpatient rates (per 100,000 individuals, 1998–2019) for ST Elevation Myocardial Infarction (STEMI) and Non-ST Elevation Myocardial Infarction (NSTEMI) (STEMI / NSTEMI (I21)), Subsequent myocardial infarctions (Subs. myoc. infarc. (I22)), Heart Failure (Heart failure (I50)), Stroke (Stroke (I61)). *P*-values are reported in each graph. Significant differences with *p*-value < 0.05 are marked by * and with *p*-value < 0.01 are marked by **

**Fig. 4:**
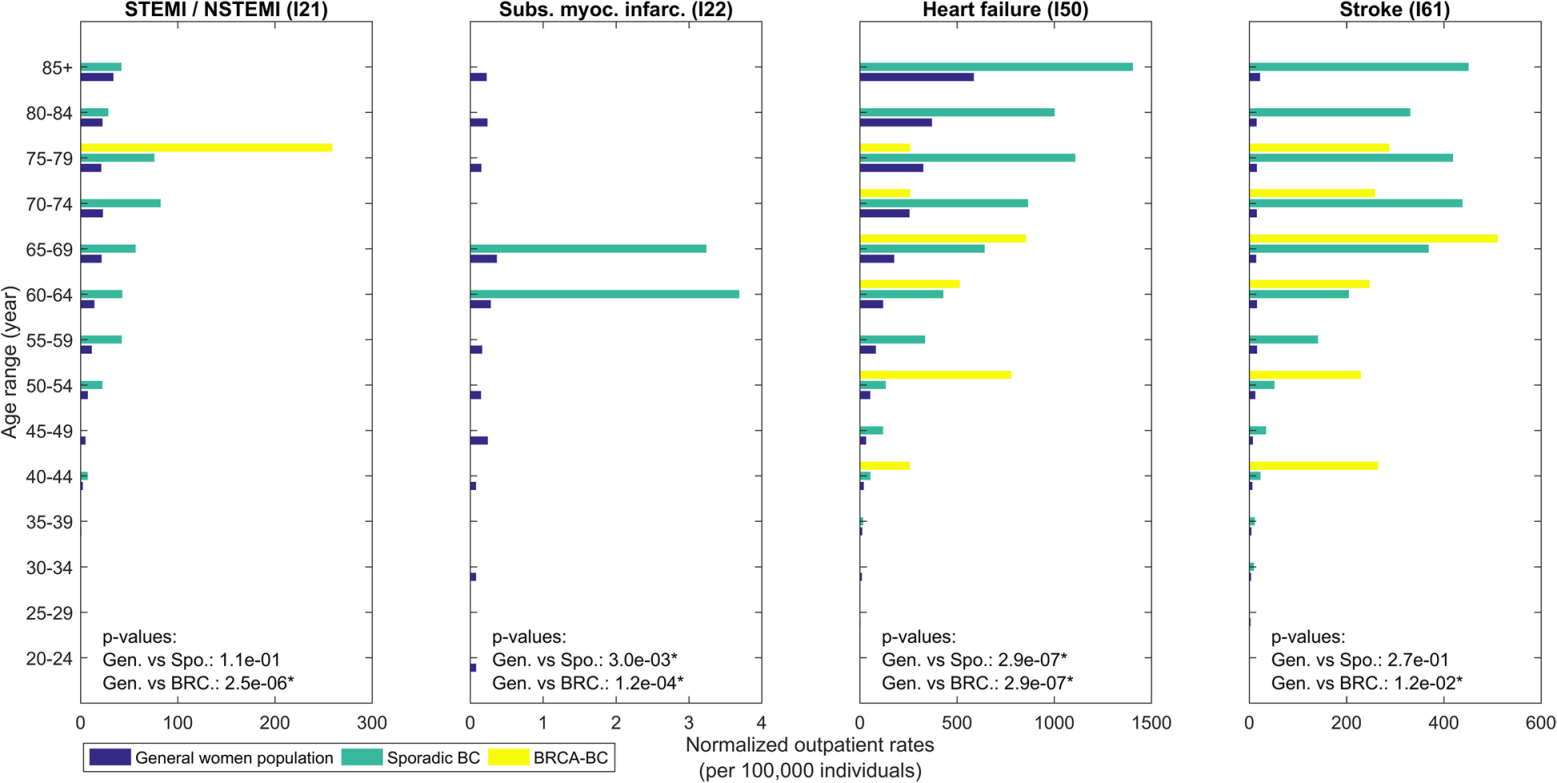
The distribution of normalized outpatient rates (per 100,000 individuals, 1998–2019) for ST Elevation Myocardial Infarction (STEMI) and Non-ST Elevation Myocardial Infarction (NSTEMI) (STEMI / NSTEMI (I21)), Subsequent myocardial infarctions (Subs. myoc. infarc. (I22)), Heart Failure (Heart failure (I50)), Stroke (Stroke (I61)). *P*-values are reported in each graph. Significant differences with *p*-value < 0.05 are marked by * and with *p*-value < 0.01 are marked by **

Figures 3 and 4 detail MACE’s age distributions and prevalence rates; the corresponding Figures for other CVDs are provided in the supplementary information (Figures S1, S1b, S2a, S2b).

Compared to the general population, Sporadic-BC patients demonstrated significantly elevated rates of several cardiovascular conditions. Inpatient HF rates significantly (*p* < 0.01) peaked at approximately 4,000 per 100,000 in Sporadic-BC patients, compared to 2,000 per 100,000 in the general population (Fig. 3). Outpatient HF rates were similarly significantly (*p* < 0.01) elevated at 1,500 per 100,000 compared to 1,000 per 100,000 (Fig. 4). STEMI/NSTEMI rates for Sporadic-BC patients were also significantly (*p* < 0.01) higher at 600 per 100,000 compared to 200 per 100,000 in the general population (Fig. 3). Stroke rates peaked at 2,000 per 100,000 in the inpatient setting but did not differ substantially from general population rates in most age groups. Supplementary Figures S1a and S1b further highlight disparities in angina pectoris and AF rates, which were consistently higher in Sporadic-BC patients compared to the general population.

In contrast, BRCA-BC patients generally exhibited lower rates of CVD across all age groups. However, HF rates in younger BRCA-BC patients (ages 40–49) approximated those in the general population, reaching ~ 200 per 100,000 (Fig. 3). Supplementary Figures S1a and S1b emphasize conditions such as angina pectoris and AF, where Sporadic-BC patients consistently exhibited higher rates. Similarly, Supplementary Figures S2a and S2b illustrate disparities in outpatient rates for procedures like PCI, with Sporadic-BC patients showing markedly higher occurrences than BRCA-BC patients.

## Discussion

This descriptive study provides a comprehensive analysis of cardiovascular morbidity and risk factors among women with Sporadic-BC and BRCA-BC over 25 years in the Stockholm-Gotland region. The findings underscore distinct cardiovascular profiles between these groups, emphasizing the importance of tailored cardio-oncology care.

Sporadic-BC patients exhibited a higher prevalence of CVD compared to BRCA-BC patients, both before and after BC diagnosis (26.4% vs. 9.8%). The median age at first CVD diagnosis was later in Sporadic-BC patients (74 years) than in BRCA-BC patients (62 years). The longer median time to first CVD after BC diagnosis in BRCA-BC patients (10.9 years vs. 6.2 years in Sporadic-BC) reflects their younger age at BC diagnosis [5, 22]. These results are consistent with findings from Abdel-Qadir et al., who reported elevated risks of HF and other cardiovascular hospitalizations following early-stage BC treatment, underscoring the impact of cancer therapies on cardiovascular morbidity [23].

In this study, we differentiated between inpatient and outpatient CVD cases, providing insights into cardiovascular complications. Inpatient cases requiring hospitalization represented acute and severe complications, while outpatient cases primarily reflected chronic, managed conditions. These findings align with previous studies emphasizing the severity of inpatient events, such as HF, stroke, and MI, in BC survivors [14, 23–25].

This study defined MACEs as HF, stroke, and MI. Among these, HF was the most frequent, with inpatient rates of 8.3% in Sporadic-BC and 2.7% in BRCA-BC patients. Stroke was observed in 4.3% of Sporadic-BC and 1.6% of BRCA-BC patients, while MI (including STEMI, NSTEMI, and subsequent MI) occurred in 3.2% and 1.1% of these groups, respectively. Compared to the general female population in Stockholm, BC patients exhibited markedly higher rates of HF, stroke, and MI across all age groups and settings. This trend underscores the broader cardiovascular impact of BC diagnosis and treatment. Inpatient HF rates were higher in Sporadic-BC patients compared to the general population, indicating an increased burden of HF requiring hospitalization. These findings underscore the substantial cardiovascular burden associated with BC, aligning with Paterson et al. (2022), who reported significantly elevated CVD risks in BC survivors compared to the general population [25].

Stroke was the second most frequent MACE in BC patients, with a higher prevalence in Sporadic-BC patients across treatment groups. For instance, in the absence of systemic therapy, stroke occurred in 787 Sporadic-BC patients versus 4 BRCA-BC patients, with a median age of 80 and 62.5 years, respectively. Our data showed varying stroke occurrence rates based on received endocrine therapy and chemotherapy, highlighting the impact of treatment regimens on cardiovascular outcomes [24]. Additionally, AF, a known contributor to ischemic stroke, was notably prevalent in both BC groups [26]. The increased prevalence of AF in both groups is consistent with findings by Ay et al., who highlighted a higher risk of AF in cancer patients compared to non-cancer controls, emphasizing the need for integrated cardio-oncology management strategies [27]. Our study focused on clinically significant arrhythmias, such as AF, to highlight their contribution to stroke risk. The exclusion of non-specific arrhythmias, including sinus tachycardia, ensured that the analysis was relevant to cardiovascular morbidity and required intervention.

Outpatient cases predominantly represented chronic, managed cardiovascular conditions, such as hypertension, which increased after BC diagnosis to 22.9% in Sporadic-BC patients and 12.1% in BRCA-BC patients. These findings align with previous studies showing heightened hypertension prevalence in BC survivors, often attributed to age, systemic therapies, and cancer-related stressors [23, 24]. However, the lower baseline and post-diagnosis hypertension rates in BRCA-BC patients may reflect their younger age at diagnosis and reduced pre-existing comorbidities, consistent with other studies of BRCA mutation carriers [28]. Other CVRFs, including diabetes mellitus and obesity, were also more prevalent in Sporadic-BC patients post-diagnosis, reflecting their older median age and higher baseline comorbidity burden.

Ischemic heart disease, including MI and angina, was observed more frequently in Sporadic-BC patients compared to BRCA-BC patients, with MI rates of 3.2% in Sporadic-BC versus 1.1% in BRCA-BC and angina rates of 2.9% in Sporadic-BC versus 0.7% in BRCA-BC. STEMI and NSTEMI were observed exclusively in Sporadic-BC patients treated with radiotherapy or no systemic therapy. For example, STEMI occurred in 10 Sporadic-BC patients who underwent radiotherapy (median age: 69.5 years) but was absent in BRCA-BC patients. This discrepancy highlights differences in underlying cardiovascular risks and treatment exposures between these groups. This increased prevalence in Sporadic-BC patients may reflect their older age, higher baseline CVRFs, and greater exposure to radiotherapy (34.4% vs. 26.9%). Radiotherapy, particularly to the left chest, has been identified as a risk factor for ischemic heart disease due to incidental exposure to cardiac structures. Bergom et al. highlighted that despite advancements in cardiac-sparing techniques, radiotherapy remains a contributor to cardiovascular morbidity [29].

HF was the most common CVD across both groups, with higher rates in Sporadic-BC patients across all treatment regimens. Sporadic-BC patients who did not receive systemic therapy had HF rates of 5.3% (1,742 patients), compared to 2.5% (11 patients) in BRCA-BC. BRCA-BC patients, however, experienced HF at younger ages (median: 66 years vs. 81 years in Sporadic-BC). These findings align with those of Brauer et al., who emphasized the impact of anthracyclines and HER2 inhibitors on cardiovascular outcomes in BC patients, particularly in BRCA-related genetic vulnerabilities [7].

The disparities in cardiovascular morbidity between BC subtypes highlight the need for tailored management strategies. BRCA-BC patients were more likely to receive neo-adjuvant chemotherapy (85.4% vs. 8.4%) and adjuvant chemotherapy (31.3% vs. 18.4%) than Sporadic-BC patients, reflecting their younger age and aggressive cancer profiles. They also had a higher proportion receiving anthracycline-containing regimens (28.8% vs. 16.5%). Conversely, Sporadic-BC patients more frequently underwent endocrine therapy (43.0% vs. 26.0%) and radiotherapy (34.4% vs. 26.9%), consistent with their older age and differing tumor biology. Use of HER2 inhibitors was rare in both groups but slightly more common in Sporadic-BC patients (5.9% vs. 1.8%). BRCA-BC patients require early cardiovascular screening and proactive management due to their younger age at onset and exposure to systemic chemotherapies. In contrast, Sporadic-BC patients benefit from comprehensive management of pre-existing CVRFs to address their higher overall burden. HF, the most frequently reported MACE across all groups, underscores the importance of integrating cardiovascular risk assessments into BC survivorship care, particularly for high-risk groups. These findings align with ESC cardio-oncology guidelines advocating multidisciplinary approaches to optimize outcomes in cancer survivors [14].

### Study limitations

This study has several limitations inherent to register-based research. The retrospective nature of the data introduces potential biases, including missing data, misclassification of ICD codes, and inconsistent coverage across years, which may impact the results. Global longitudinal strain (GLS) measurements, a sensitive marker for subclinical cardiac dysfunction, were not included, limiting insights into early cardiovascular changes. Additionally, data on smoking habits, socioeconomic factors, and heredity were unavailable, further restricting the analysis.

The exclusion of primary care data may underrepresent the prevalence of chronic cardiovascular conditions managed outside hospital settings, limiting the findings’ generalizability. Furthermore, this descriptive study does not employ regression or causal inference analyses to adjust for potential confounders, such as age, treatment regimens, or comorbidities. The observed associations between BC treatments and cardiovascular outcomes are observational and warrant further investigation in prospective or matched cohort studies.

The comparison with the general female population of Stockholm, which included a small number of BC patients, provides only crude estimates. Additionally, the exclusion of Gotland, a region with a small population (~ 60,000), is unlikely to have significantly influenced these comparisons. Changes in diagnostic criteria over the past decades may also have affected disease incidence trends within the cohort. Finally, the small number of cardiovascular events in BRCA-BC patients reduced statistical power, warranting cautious interpretation of the results.

## Conclusion

The distinct cardiovascular profiles of Sporadic-BC and BRCA-BC patients highlight the need for personalized care strategies. BRCA-BC patients require early screening and proactive management due to their younger age at onset. In contrast, Sporadic-BC patients face a higher overall cardiovascular burden, necessitating comprehensive management of comorbidities. Integrating cardiovascular care into survivorship plans is critical to improving outcomes. Future research should explore the genetic and treatment-related mechanisms underlying cardiovascular risks to refine care strategies for these vulnerable populations.

### Clinical perspective

These findings align with ESC cardio-oncology guidelines, emphasizing the importance of initial risk stratification and routine follow-up for BC survivors, particularly those with BRCA1/2 mutations. The study highlights the critical need for vigilant cardiovascular monitoring and proactive management in BC patients, with tailored strategies for BRCA mutation carriers to address their unique risks. Incorporating comprehensive cardiovascular risk assessments into survivorship care plans can help mitigate CVD risks and enhance patient outcomes. Further research should focus on elucidating the mechanisms driving the increased cardiovascular risk in BRCA mutation carriers and developing targeted interventions to address these challenges effectively.

## Supplementary Information

Supplementary Material 1.

Supplementary Material 2.

## Data Availability

The datasets generated and/or analyzed during the current study are not publicly available because of Swedish laws and regulations, but they are available from the corresponding author upon reasonable request.

## Abbreviations

BC: Breast Cancer
CVD: Cardiovascular Disease
CVRF: Cardiovascular Risk Factor
CTR-CVT: Cancer Therapy-Related Cardiovascular Toxicity
NPR: National Patient Register
ICD-10: International Classification of Diseases, 10th Revision
IQR: Interquartile Range
BRCA1/2: BRCA1 and BRCA2 Genes
CAD: Coronary Artery Disease
MACE: Major Adverse Cardiovascular Events
HF: Heart Failure
MI: Myocardial Infarction, including STEMI, NSTEMI, or subsequent infarctions; I21.0–I21.4
STEMI: ST-Elevation Myocardial Infarction
NSTEMI: Non-ST-Elevation Myocardial Infarction
AF: Atrial Fibrillation/Flutter
HER2: Human Epidermal Growth Factor Receptor 2
