# Supplementary material for: Cardiovascular health in breast cancer patients: insight on BRCA1/2 mutations impact
Source: Cardiooncology. 2025 Jan 21;11:5. doi: 10.1186/s40959-025-00302-z (PMC11749121; doi:10.1186/s40959-025-00302-z)
Supplement: Supplementary file 1 — Supplementary Material 1. [file 40959_2025_302_MOESM1_ESM.docx]

**Supplemental Appendix**

**Cardiovascular Health in Breast Cancer Patients: insight on BRCA1/2 Mutations Impact.**

Table of Contents

[Supplemental Method 2](#_Toc186448544)

[Breast Cancer Treatment Evolution (1995-2020) 2](#_Toc186448545)

[Supplemental Tables 3](#_Toc186448546)

[Supplemental Table 1. Swedish population registers are used to obtain information on cohort 3](#_Toc186448547)

[Supplemental Tabel 2. International Classification of Diseases (ICD-10) codes define cardiovascular events and comorbidity conditions. 5](#_Toc186448548)

[Supplemental Figure Legends 8](#_Toc186448549)

# Supplemental Method

## Breast Cancer Treatment Evolution (1995-2020)

In Sweden, the Swedish Breast Cancer Group is pivotal in establishing evidence-based national guidelines for BC treatment (1). These guidelines are regularly updated to reflect significant changes in medical evidence, and Swedish healthcare professionals are obligated to adhere to them.

Before April 2002, the standard adjuvant treatments for early-stage BC included anthracycline-containing chemotherapy (FEC - 5-fluorouracil, epirubicin, cyclophosphamide) or non-anthracycline-containing chemotherapy (CMF - cyclophosphamide, 5-fluorouracil, and methotrexate), both administered intravenously at three-week intervals. However, after April 2002, the recommended therapy shifted towards anthracycline-containing chemotherapy and regimens that included taxanes. Furthermore, since 2005, patients in the Stockholm-Gotland region with HER2-positive early BC have been recommended trastuzumab treatment instead of no treatment option.

Radiation therapy (RT) to the breast became a standard practice after breast-conserving surgery and occasionally to the chest wall after mastectomy. Patients with lymph node-positive disease received loco-regional RT (45-50 Gray), aiming to limit the mean heart dose to less than 10%.

Adjuvant antiestrogen therapy, such as tamoxifen or aromatase inhibitors, was administered after chemotherapy and RT to women with an estrogen receptor-positive tumor or unknown hormone receptor status, depending on their menopausal status. For premenopausal women, tamoxifen treatment extended to 5 years until 2015, after which it increased to 10 years. Tamoxifen, a selective estrogen receptor modulator (SERM), prevented estrogen binding. In premenopausal women with hormone-positive BC at high risk of relapse, oophorectomy (medical, radiological, or surgical) was combined with tamoxifen rather than tamoxifen alone.

Postmenopausal women received five years of tamoxifen or aromatase inhibitors, which blocked estrogen production. In some instances, they followed a regimen of 2 years of tamoxifen followed by three years of aromatase inhibitors (switch treatment).

In early-stage BC, systemic oncological treatment can be given before surgery (neoadjuvant therapy) or after surgery (adjuvant therapy), depending on the tumor’s characteristics.

**Reference:**

1. Swedish Breast Cancer Group [Svenska Bröstcancergruppen] (2020). National Guideline for Breast cancer [Available from: <https://www.swebcg.se/vardprogram/> Accessed 20 January 2023.

# Supplemental Tables

## **Supplemental Table 1**. Swedish population registers are used to obtain information on cohort

| **Supplementary Table 1.** Swedish population registers used to obtain information on cohort | | | |
| --- | --- | --- | --- |
| **Register** | **Description** | **Years** | **Information retrieved** |
| Swedish National Cancer Register | Started in 1958 and includes data on histological type, site, date of diagnosis, eventual date and cause of death, with a coverage rate of 96%.^1,2^ | 1958-2019 | date of BC diagnosis, age at diagnosis, tumor characteristics, treatment details for BC, date of relapse |
| Swedish National Quality Register for BC | A population-based register, with information on tumor characteristics, treatment, and relapse occurrence in patients diagnosed with invasive BC since 2008, with a coverage rate of 99%.^3^ | 2008-2019 |  |
| Regional quality registers for BC, for region Stockholm-Gotland | Prior to 2008, data on tumor characteristics, treatment details and relapse occurrence for all cases of invasive BC were reported to the six regional quality registers. | 1995-2007 |  |
| The Swedish National Patient Registry | Encompasses both inpatient and outpatient healthcare. It captures data on patients who have been hospitalised (inpatients) and those who have received medical care from healthcare providers without hospital admission (outpatients) for diagnosis, treatment, or follow-up.  The inpatient registry has been operational since 1964, while the outpatient registry started in 2003. These registries are essential to Sweden's healthcare information infrastructure vital in monitoring and improving healthcare services nationwide. ^5-8^ | 1964-2020 (Inpatinet) and 2003-2020 (Outpatinet) | Date of comorbidities and cardiovascular diseases. |
| Abbreviations: BC, breast cancer. | | | |
| References:   1. Socialstyrelsen. Swedish National Cancer Register. Available from: https://www.socialstyrelsen.se/en/statistics-and-data/registers/register-information/swedish-cancer-register/. [Accessed 1^st^ June 2023] 2. Barlow L, Westergren K, Holmberg L, et al: The completeness of the Swedish Cancer Register: a sample survey for year 1998. Acta Oncol 48:27-33, 2009 3. Lofgren L, Eloranta S, Krawiec K, Asterkvist A, Lonnqvist C, Sandelin K, et al. Validation of data quality in the Swedish National Register for Breast Cancer. BMC Public Health. 2019;19(1):495. 4. Brooke HL, Talback M, Hornblad J, Johansson LA, Ludvigsson JF, Druid H, et al. The Swedish cause of death register. Eur J Epidemiol. 2017;32(9):765-73. 5. Socialstyrelsen. Swedish National Patient Registry. Available from: <https://www.socialstyrelsen.se/en/statistics-and-data/registers/national-patient-register/> [Accessed 1^st^ June 2023] 6. Ludvigsson JF, Andersson E, Ekbom A, et al: External review and validation of the Swedish national inpatient register. BMC Public Health 11:450, 2011 7. Johansson LA, Westerling R: Comparing Swedish hospital discharge records with death certificates: implications for mortality statistics. International Journal of Epidemiology 29:495-502, 2000 8. Ludvigsson JF, Almqvist C, Bonamy AK, Ljung R, Michaelsson K, Neovius M, et al. Registers of the Swedish total population and their use in medical research. Eur J Epidemiol. 2016;31(2):125-36. | | | |

## Supplemental Tabel 2. International Classification of Diseases (ICD-10) codes define cardiovascular events and comorbidity conditions.

| Disease | ICD-10 | ICD-9 | ICD-8 | ICD-7 | ICD-6 |
| --- | --- | --- | --- | --- | --- |
| Risk factors for CVD |  |  |  |  |  |
| Diabetes mellitus | E10  E11 | 250.x | 250.x | 260 | 260 |
| Hyperlipidemia | E78 | 272.x | 272.x | 289.0 | 289.0 |
| Chronic kidney disease | N18 | 585 | 582 | 592.x | 592 |
| Acute/unspecified renal failure | N17  N19 | 584  586 | 580  583 | 590.x  593.x | 590  593 |
| Obesity (BMI>30 kg/m2) | E65  E66 | 278.x | 277 | 287 | 287 |
| Hypertension | I10 | 401.x | 401 | 444  445 | 444  445 |
| Cardiovascular Event |  |  |  |  |  |
| Angina Pectoris | I20 | 413.x | 413.x | 420.2 |  |
| Myocardial Infarction | | | | | |
| ST Elevation MI (STEMI) | I21.0 I21.1  I21.2 | 410.0-  410.6  410.8  410.9 | 410.x | 420.1 |  |
| Non-ST Elevation MI (NSTEMI) | I21.4 | 410.7 | 410.x | 420.1 |  |
| Subsequent Myocardial Infarction | I22 | 410.02  410.12  410.22  410.32 | 411 | 420.1 |  |
| Atrial fibrillation and  Atrial Flutter | I48  I48 | 427.3  427.3 | 427.4  427.4 | 433.12  433.13 | 433.1  433,1 |
| Complete Heart Block | | | | | |
| Atrioventricular Block 3^rd^ degree (complete) | I44.2 | 426.0 | 427.3 | 433.02 | 433.0 |
| Ventricular Arrhythmias | | | | | |
| Ventricular tachycardia | I47.2 | 427.1 | 427.9 | 433.11 | 433.1 |
| Ventricular fibrillation/flutter | I49.0 | 427.4 | 427.6 | 433.11 |  |
| Percutaneous Coronary Revascularization (PCI): | | | | | |
| Coronary stent implant | Z95.5 | V45.82 | - | - | - |
| PCI procedures | Z98.61  0270  0271  0272  0273 | 36.06  36.07 |  |  |  |
| Coronary Artery Bypass Grafting (CABG) | Z95.1  0210  0211  0212  0213 | V45.81  36.1x |  |  |  |
| Pacemaker Implantation | Z95.0  02HK3J  02HK3M  02HK0J | V45.01  37.78 | Y29 | - | - |
| Resynchronization Therapy or Implantable Cardioverter Defibrillator (CRT/ICD) Implantation | Z95.0  Z95.810  02HK0K  02HK3K  02HL0J  02HL0K  02HL3J  02HL3K | V45.02  00.50  00.51  00.53  00.54  37.94 |  |  |  |
| Heart Failure Hospitalization (Inpatient registery) | I50 | 428.x | 427.0  427.1  428 | 434.1  434.2 | 434.1  434.2 |
| Heart Failure Outpatient registry | I50 | 428.x | 427.0  427.1  428 | 434.1  434.2 | 434.1  434.2 |
| Stroke | I63  I64 | 433.x  434.x | 432.x  433.x  434.x | 332.x | 332 |
| Claudication | I73.9 | 443.9 | 443.9  458.9 | 453.33 | 453.3 |
| Peripheral Arterial Revascularization | Z95.82 | V43.4 |  |  |  |
| Abbreviations: ICD-10 = International Coding of Disease classification. ICD codes. | | | | | |

# Supplemental Figure legends

**Figure S1a.** The distribution of normalized inpatient rates (per 100,000 individuals, 2001-2019) for Angina pectoris (Angina pectoris (I20)), Complete heart block (Comp. heart block (I44)), and Ventricular tachycardia (Vent. tachycardia (I47)), and Atrial fibrillation or atrial flutter (Atrial fibrill. / flut. (I48)). Significant differences with p-value < 0.01 are marked by **.

**Figure S1b.** The distribution of normalized inpatient rates (per 100,000 individuals, 2001-2019) for Ventricular fibrillation/flutter (Vent. fibrill. / flut. (I49)), Claudication (Claudication (I70)), and Percutaneous coronary revascularization (PCI) group (PCI (Z95)). P-values are reported in each graph. Significant differences with p-value < 0.01 are marked by **.

**Figure S2a**. The distribution of normalized outpatient rates (per 100,000 individuals, 2001-2019) for Angina pectoris (Angina pectoris (I20)), Complete heart block (Comp. heart block (I44)), and Ventricular tachycardia (Vent. tachycardia (I47)), and Atrial fibrillation or atrial flutter (Atrial fibrill. / flut. (I48)). Significant differences with p-value < 0.01 are marked by **.

**Figure S2b.** The distribution of normalized outpatient rates (per 100,000 individuals, 2001-2019) for Ventricular fibrillation/flutter (Vent. fibrill. / flut. (I49)), Claudication (Claudication (I70)), and Percutaneous coronary revascularization (PCI) group (PCI (Z95)). P-values are reported in each graph. Significant differences with p-value < 0.05 are marked by * and with p-value < 0.01 are marked by **.
