# Supplementary figures and images for: Cardiovascular health in breast cancer patients: insight on BRCA1/2 mutations impact
Source: Cardiooncology. 2025 Jan 21;11:5. doi: 10.1186/s40959-025-00302-z (PMC11749121; doi:10.1186/s40959-025-00302-z)

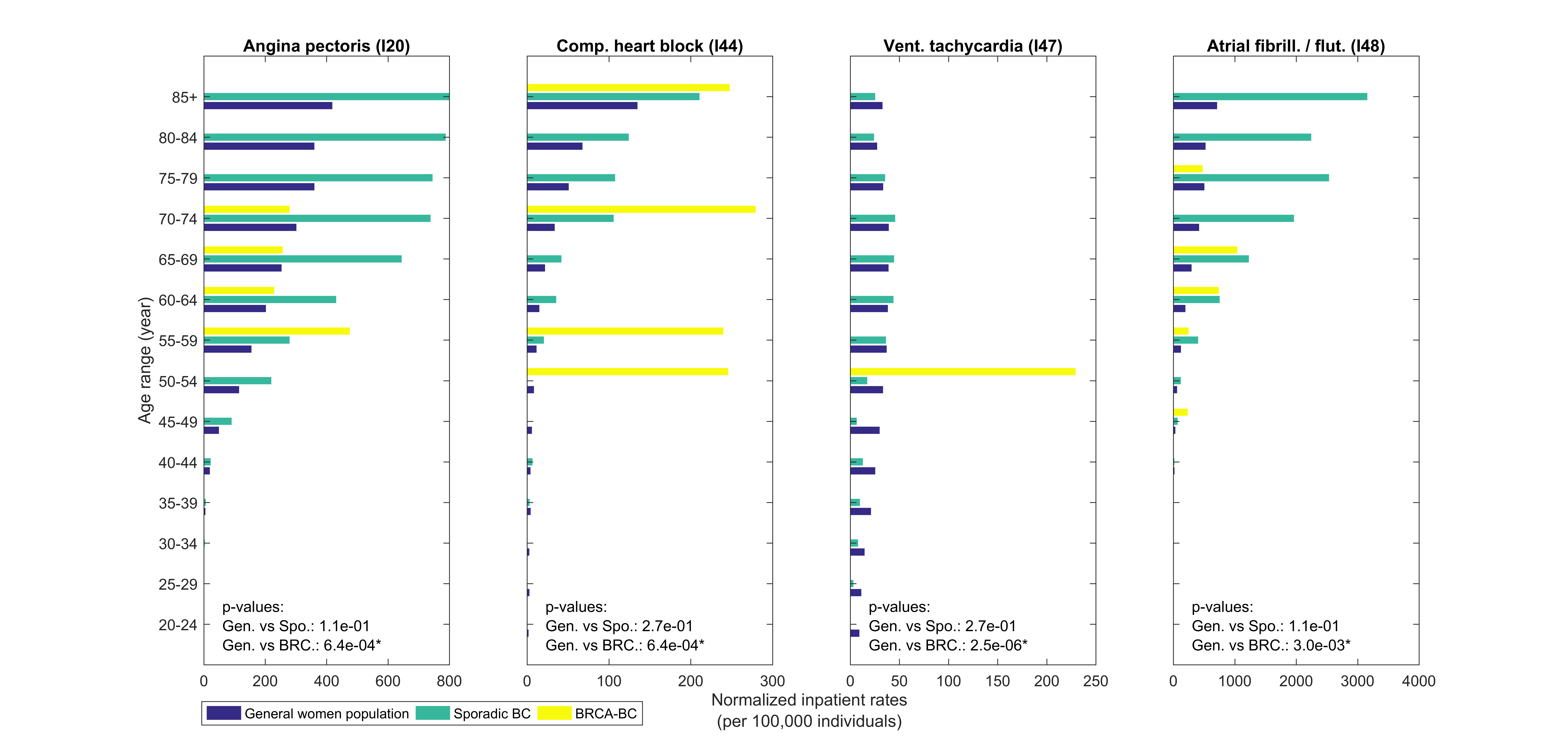

Supplement: Supplementary file 2 — Supplementary Material 2. [file 40959_2025_302_MOESM2_ESM.zip › Figure_S1a.tif]

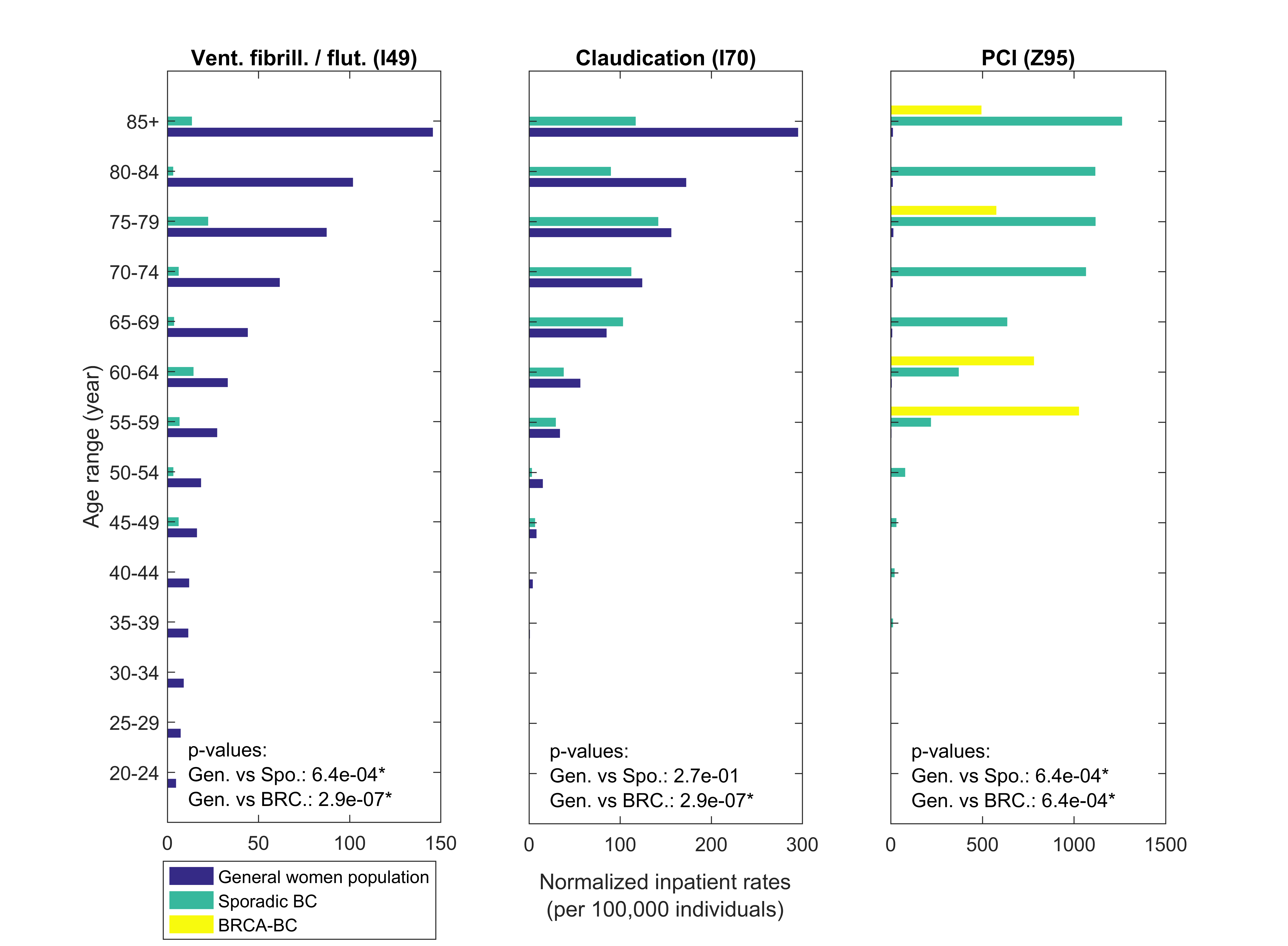

Supplement: Supplementary file 2 — Supplementary Material 2. [file 40959_2025_302_MOESM2_ESM.zip › Figure_S1b.tif]

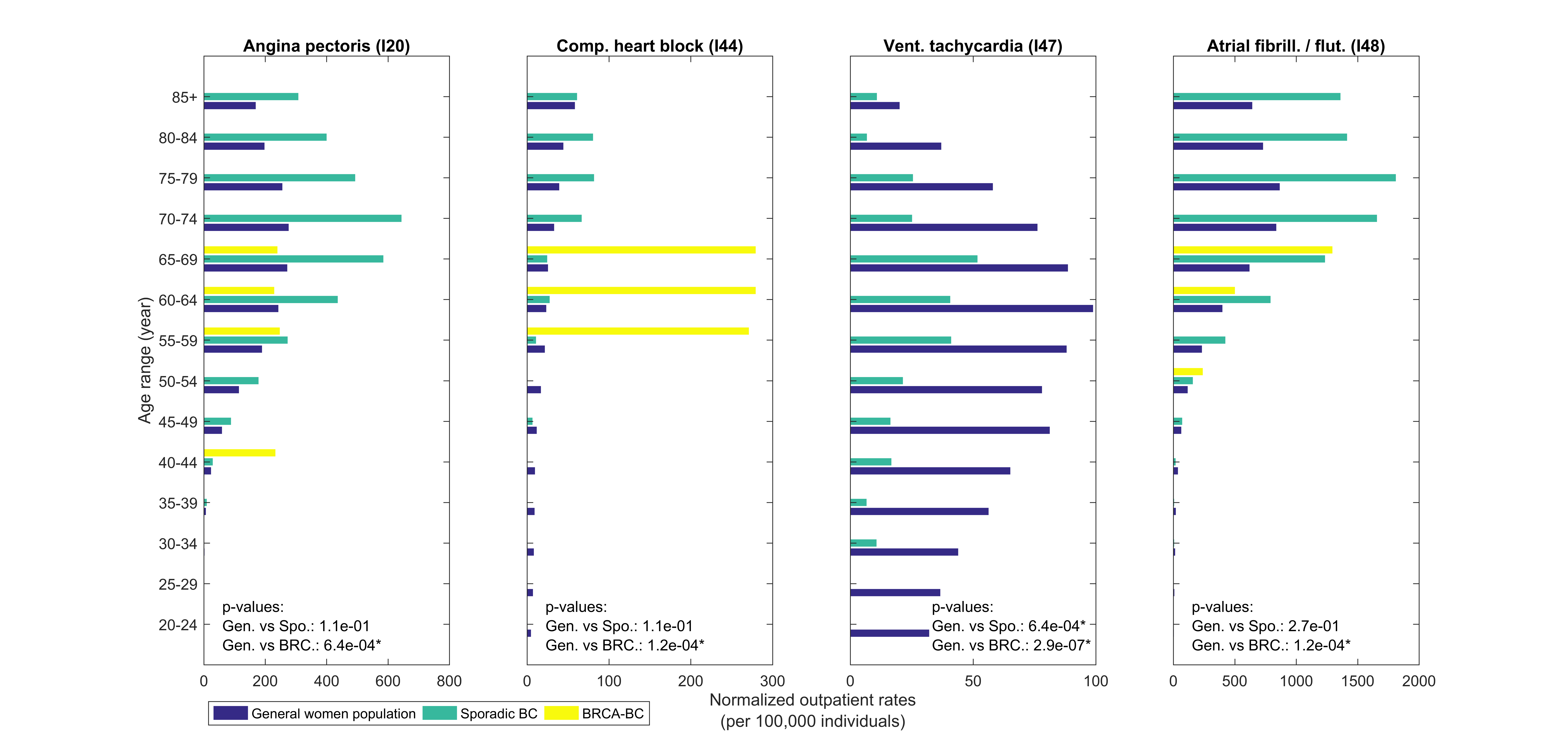

Supplement: Supplementary file 2 — Supplementary Material 2. [file 40959_2025_302_MOESM2_ESM.zip › Figure_S2a.tif]

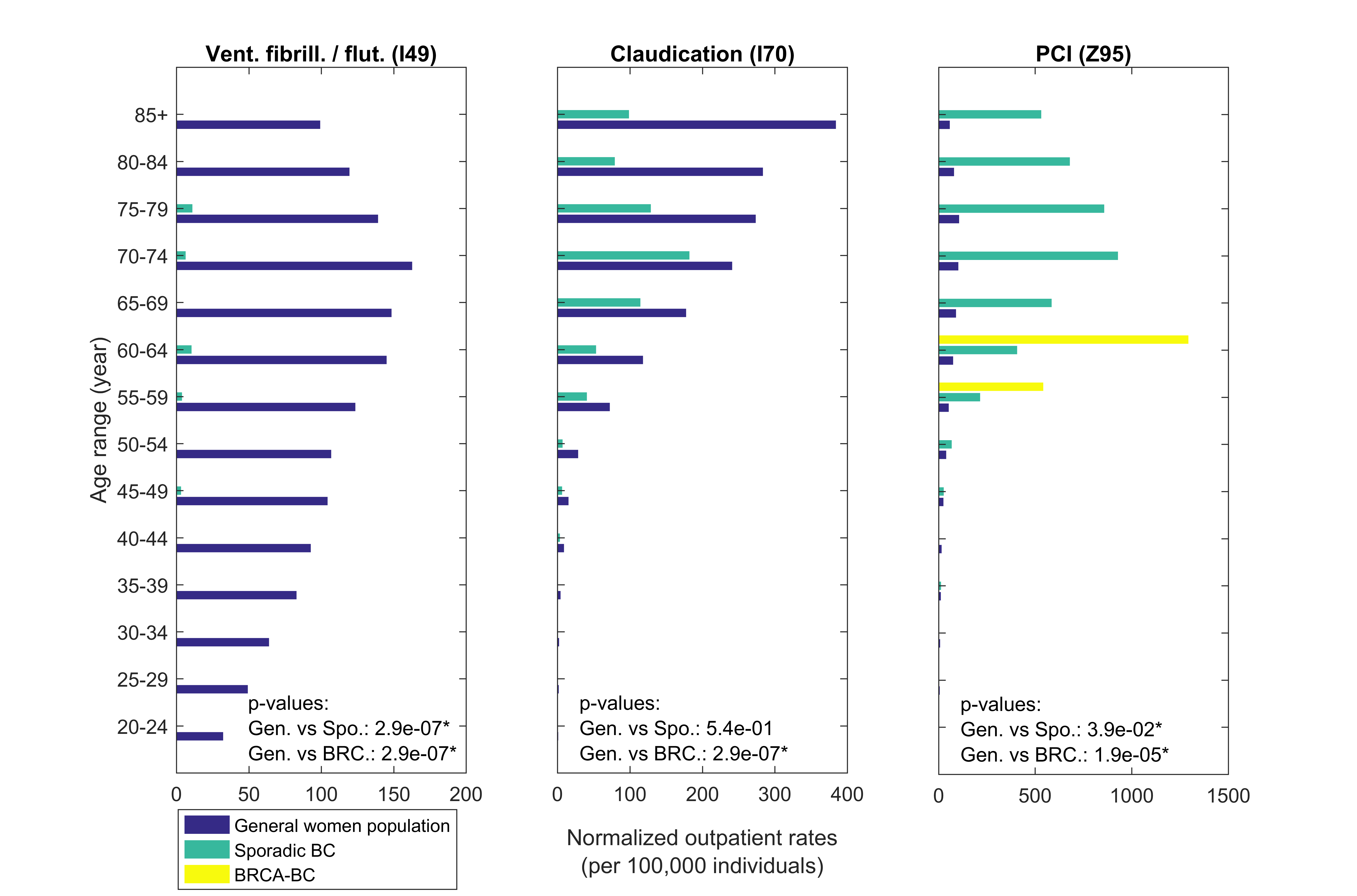

Supplement: Supplementary file 2 — Supplementary Material 2. [file 40959_2025_302_MOESM2_ESM.zip › Figure_S2b.tif]
